# MnO_2_ and roflumilast-loaded probiotic membrane vesicles mitigate experimental colitis by synergistically augmenting cAMP in macrophage

**DOI:** 10.1186/s12951-024-02558-6

**Published:** 2024-05-28

**Authors:** Chengjun Song, Jiamin Wu, Jinhui Wu, Fangyu Wang

**Affiliations:** 1 State Key Laboratory of Pharmaceutical Biotechnology, Medical School, Nanjing University, Nanjing, 210093 China; 2 Department of Gastroenterology and Hepatology, Nanjing Jinling Hospital, Affiliated Hospital of Medical School, Nanjing University, Nanjing, 210002 China; 3 Institution of Drug R&D, Nanjing University, Nanjing, 210093 China; 4 Chemistry and Biomedicine Innovation Center, Nanjing University, Nanjing, 210093 China

**Keywords:** Membrane vesicles, Roflumilast, MnO_2_, cAMP, Macrophage, Ulcerative colitis

## Abstract

**Background:**

Ulcerative colitis (UC) is one chronic and relapsing inflammatory bowel disease. Macrophage has been reputed as one trigger for UC. Recently, phosphodiesterase 4 (PDE4) inhibitors, for instance roflumilast, have been regarded as one latent approach to modulating macrophage in UC treatment. Roflumilast can decelerate cyclic adenosine monophosphate (cAMP) degradation, which impedes TNF-α synthesis in macrophage. However, roflumilast is devoid of macrophage-target and consequently causes some unavoidable adverse reactions, which restrict the utilization in UC.

**Results:**

Membrane vesicles (MVs) from probiotic *Escherichia coli* Nissle 1917 (EcN 1917) served as a drug delivery platform for targeting macrophage. As model drugs, roflumilast and MnO_2_ were encapsulated in MVs (Rof&MnO_2_@MVs). Roflumilast inhibited cAMP degradation via PDE4 deactivation and MnO_2_ boosted cAMP generation by activating adenylate cyclase (AC). Compared with roflumilast, co-delivery of roflumilast and MnO_2_ apparently produced more cAMP and less TNF-α in macrophage. Besides, Rof&MnO_2_@MVs could ameliorate colitis in mouse model and regulate gut microbe such as mitigating pathogenic *Escherichia–Shigella* and elevating probiotic *Akkermansia*.

**Conclusions:**

A probiotic-based nanoparticle was prepared for precise codelivery of roflumilast and MnO_2_ into macrophage. This biomimetic nanoparticle could synergistically modulate cAMP in macrophage and ameliorate experimental colitis.

**Graphical Abstract:**

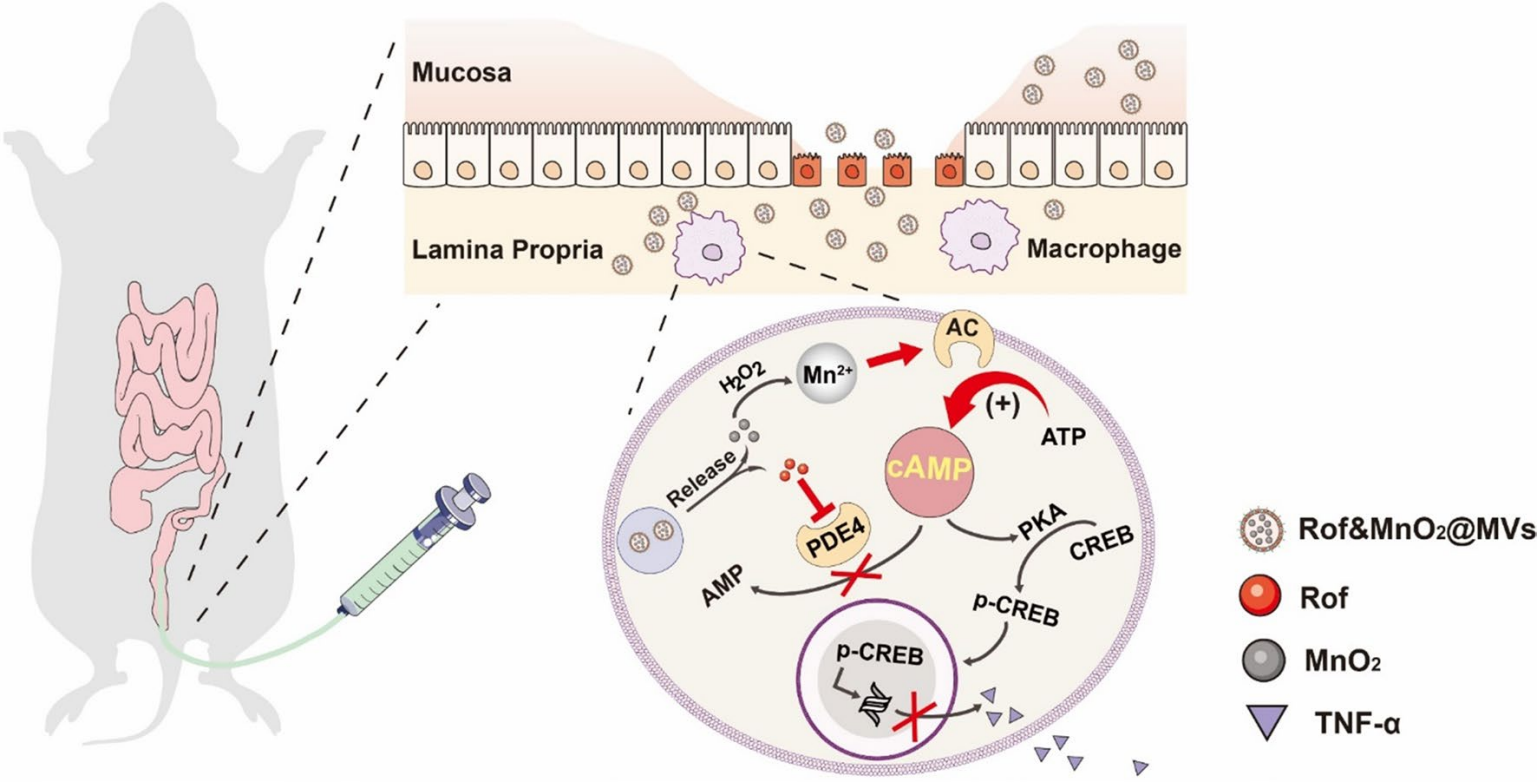

**Supplementary Information:**

The online version contains supplementary material available at 10.1186/s12951-024-02558-6.

## Introduction

Ulcerative colitis (UC) is one chronic and relapsing inflammatory bowel disease with unclear etiology, whose pathological manifestations include mucosa and submucosa damage, immunocyte infiltration and crypt abscess [1, 2]. The morbidity of UC has been augmenting recently, which aggravates the global health burden. Moreover, patients with UC have more risk of colon and rectum carcinoma [3]. Biopsy from UC patients revealed that more macrophage infiltration in colon tissue than healthy volunteers [4]. Previous research has uncovered that macrophage serves as a trigger to elicit the downstream inflammatory cascade in the mucosa after swallowing microorganism [5–7]. Nowadays, there have been several types of drugs for modulating macrophage, including nonsteroidal anti-inflammatory drugs (NSAIDs), cortisol, immunosuppress agents and biological agents [5, 8, 9]. Additionally, new therapeutics are investigated, for instance, phosphodiesterase 4 (PDE4) inhibitors.

Some PDE4 inhibitors have been under investigation in both animal models and clinical trials of UC, such as roflumilast (Daliresp®) and apremilast (Otezla®). PDE4 is the significant type of PDE family in immunocytes [10–12], which is responsible for degrading cyclic adenosine monophosphate (cAMP) in the cytoplasm [13]. It has been reported that high level of cAMP in the cytoplasm can downregulate TNF-α generation in macrophage [10, 14–16]. Previous research revealed that PDE4 inhibitors elevated cAMP and reduced TNF-α in animal colon tissue after oral administration [17–19]. Another one illustrated that apremilast could improve clinical and endoscopic manifestations among patients [20]. In addition, a recent clinic trial has been launched to estimate roflumilast efficacy in UC patients [21]. However, some adverse effect is still inevitable such as diarrhea, weight loss, nausea and psychiatric events, which restrained the usage in UC management [11].

Besides PDE4 inhibitors, it has been verified that manganese ion (Mn^2+^) can increase cytosolic cAMP level. Since cAMP is generated from adenosine triphosphate (ATP) via adenylate cyclase (AC), Mn^2+^ can serve as cofactors of AC and consequently accelerate cAMP production [22–24]. A clinic investigation on trace element manifested that UC patients had less manganese than healthy people [25]. Additionally, manganese-deficient diet indeed mitigated the tolerance of experimental colitis [26]. These results implied that manganese probably possessed anti-inflammation property in UC.

Herein, it is hypothesized that PDE4 inhibitors and Mn^2+^ can synergistically produce more cAMP in macrophage to ameliorate UC. However, some inherent properties of PDE4 inhibitors become the obstacles, such as hydrophobic, adverse effect and deficiency in macrophage-target. As a result, a vehicle is required for co-delivering PDE4 inhibitors and Mn^2+^ into macrophage precisely to circumvent these obstructions. Inspired from bacteria phagocytosis of macrophage, membrane vesicles (MVs) are regarded as the natural potential candidates.

Considering biocompatibility, MVs were obtained from probiotic *Escherichia coli* Nissle 1917 (EcN 1917). EcN 1917 (Mutaflor®) was found the same efficacy as mesalamine in a clinic trial and thus was recommended in UC management guidelines [27–29]. Similar to intact bacteria, there are multiple pathogen-associated molecular patterns (PAMPs) in the membrane, such as lipopolysaccharide, mannose and porins. These PAMPs are responsible for mediating the phagocytosis of macrophage [30]. Thus, developing biometric vectors has been an intriguing field in UC treatment [31–33]. Likewise, PAMPs render MVs intrinsic macrophage-target ability. Besides, MVs can serve as a superior vector for co-delivery of hydrophilic and hydrophobic drugs.

**Scheme 1:**
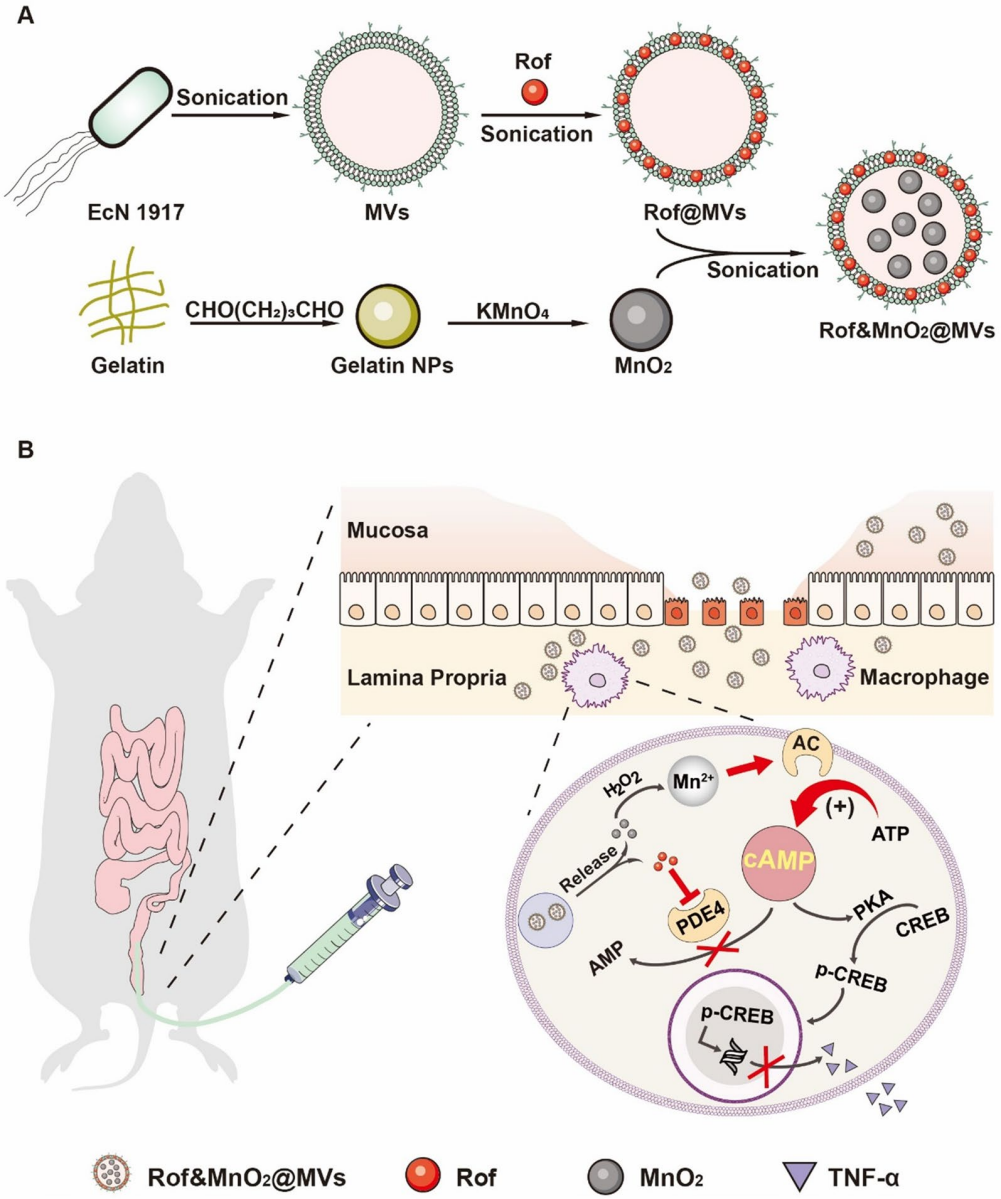
The schematic procedure of various nanoparticles preparation and the potential mechanism of UC treatment. **A** Rof@MVs, MnO_2_@MVs and Rof&MnO_2_@MVs were synthesized by sonication. Gelatin NPs, Gelatin nanoparticles. EcN, *Escherichia coli* Nissle 1917. **B** After enema, Rof&MnO_2_@MVs passed through the defective epithelial layer in the inflammatory mucosa and were swallowed by macrophage in the lamina propria. MnO_2_ was reduced into Mn^2+^ by H_2_O_2_ and Mn^2+^ bonded with adenylate cyclase (AC). Besides, roflumilast inhibited the activity of PDE4. Then roflumilast and MnO_2_ increased the concentration of cAMP, in terms of elimination and production respectively. As a second messenger, cAMP could bind with protein kinase A (PKA) to phosphorylate cAMP-response element binding protein (CREB). Phosphorylated CREB (p-CREB) entered the cell nucleus and downregulated TNF-α expression

Therefore, we constructed MVs-based nanoparticles for co-delivery of roflumilast and MnO_2_ (Rof&MnO_2_@MVs) into macrophage, in order to increase cytoplasmic cAMP synergistically. In this formulation, hydrophobic roflumilast (Rof) was encapsulated in the phospholipid bilayer and hydrophilic MnO_2_ nanoparticles lay in the core of MVs. With the biomimetic trait, MVs endowed the cargo the characterization of being devoured by the macrophage, due to PAMPs mentioned before. After uptake, the cargoes could exert their effect respectively. On the one hand, Mn^2+^, which was produced from the reaction between MnO_2_ and H_2_O_2_, bonded with AC to generate more cAMP. On the other hand, roflumilast inhibited cAMP elimination when combined with PDE4 (Scheme 1). The following results manifested that MVs-based nanoparticles were eminent vehicles for macrophage-targeted delivery. And in macrophage, there definitely existed a synergistic effect between MnO_2_ and roflumilast in augmenting the level of cAMP and thwarting TNF-α production. In a dextran sulfate sodium (DSS)-induced murine colitis model, Rof&MnO_2_@MVs demonstrated superiority of alleviating colitis over MnO_2_@MVs, Rof@MVs and 5-aminosalicylic acid (5-ASA, mesalamine, standard drug for UC).

## Materials and methods

### Materials

Gelatin (240 g Bloom), 5-aminosalicylic acid (5-ASA), fluorescein isothiocyanate and, 2′,7′-dichlorofluorescein diacetate (DCFH-DA), were purchased from Aladdin (Shanghai, China). Roflumilast, glutaraldehyde and manganese dichloride were obtained from Macklin (Shanghai, China). FITC-Dextran (average molecular weight 4kD, FD4) was purchased from Sigma-Aldrich (FD4-1G). Beta actin (β-actin) antibody (20536-1-AP), Occludin antibody (27260-1-AP), F4/80 antibody (28463-1-AP) and CoraLite594-conjugated antibody (SA00013-4) were bought from Proteintech (Wuhan, China). Phospho-CREB (Ser133) (87G3) Rabbit mAb (9198S) was obtained from Cell Signaling Technology (Shanghai, China). TNF-α ELISA kit (1217202) was bought from DAKEWE (Shenzhen, China). BCA protein kit (P0012) was purchased from Beyotime (Shanghai, China). cAMP-Glo™ Assay (V1501) was purchased from Promega. Male mice (C57BL/6J) were obtained from Comparative Medicine Centre of Yangzhou University. Other chemical reagents and kits were purchased from commercial sources.

### Extraction of MVs

MVs were prepared according to the previous research with some modifications. EcN 1917 was cultured in Luria–Bertani (LB) medium for 24 h at 37 °C. Then, the bacteria were collected by centrifugation at 4000×*g* for 15 min and homogenized via powerful sonication (3 kW) to gain MVs. Next, MVs solution was purified by centrifugation at 1500×*g* for 15 min and ultracentrifugation at 150,000×*g* for 2 h. The sediment was dispersed in deionized water by ultrasound and the concentration of MVs was determined by BCA kit. In addition, dynamic light scattering diameter analysis was performed (Figure S1).

### Preparation of gelatin nanoparticles

Gelatin nanoparticles were obtained by two-method desolvation. Namely, 0.2 g gelatin was dissolved in 20 mL of deionized water at 40 °C and then equivalent volume of 95% ethanol (v/v) was poured slowly into the solution. Next, supernatant was discarded and 60 mL 95% ethanol (v/v) was added into the gel dropwise at 40 °C. When it was finished, the solution was stirred vigorously and 0.2 mL of 25% glutaraldehyde (v/v) was added for sequent conjugation overnight. Next, the gelatin nanoparticles solution was dialyzed to remove remnant ethanol. Then, dynamic light scattering diameter analysis was performed (Figure S1). Lyophilization was performed for quantity of gelatin nanoparticles concentration.

### Preparation of MnO_2_ nanoparticles

MnO_2_ was synthesized via the reaction between potassium permanganate and gelatin (mass ratio, 1:4) for overnight. Namely, 0.45 mL of 20 mg/mL KMnO_4_ was added into 3 mL of 12 mg/mL gelatin nanoparticles with vigorous stir. Ion was eliminated by dialysis in deionized water. Then, MnO_2_ was processed for XPS, dynamic light scattering diameter analysis.

### Determination of MnO_2_

Formaldehyde oxime was prepared by mixing 10 g hydroxylamine hydrochloride with 5 mL of 35% formaldehyde and adding 95 mL H_2_O into 100 mL. 0.1 mL of MnO_2_ was dispersed in 1.9 mL HCl (1 mol/L) for 24 h reaction to generate Mn^2+^. Then, 0.1 mL of the above solution was added into 1.7 mL of NH_4_Cl–NH_3_ buffer (1 mol/L, pH 10–11). Next, 0.1 mL of formaldehyde oxime and 0.1 mL EDTA-4Na (1 mol/L) were mixed in the solution. The analysis was executed at 450 nm via ultraviolet–visible spectrophotometer (Figure S4).

### Synthesis of MVs-based nanoparticles

100 μL of 5 mg/mL roflumilast (in ethanol) was added into 6 mL of 2 mg/mL MVs and the mixture was sonicated for 8 min (350 W). Then, free Rof was removed via low-speed centrifuge to obtain Rof@MVs (Rof, 40 μg/mL). To ensure all MnO_2_ was entrapped in Rof@MVs, different concentration ratios of Mn and MVs were explored and 1:20 was chosen (Figure S2). The synthesis of MnO_2_@MVs (MnO_2_, 80 μg/mL) was by the mix of 1.5 mL of 160 μg/mL MnO_2_ and 1.5 mL of 2 mg/mL of MVs with 4-min sonication (65 W). For the preparation of Rof&MnO_2_@MVs (Rof, 20 μg/mL; MnO_2_, 80 μg/mL), 1.5 mL of Rof@MVs (Rof, 40 μg/mL) and 1.5 mL of 160 μg/mL MnO_2_ were homogenized by 4-min sonication (65 W). Then, Rof&MnO_2_@MVs were observed via SEM. For further use, these nanoparticles were lyophilized. Rof&MnO_2_@MVs (Rof, 40 μg/mL; MnO_2_, 80 μg/mL) preparation was in the similar way. 200 μL of 5 mg/mL roflumilast (in ethanol) was added into 6 mL of 2 mg/mL MVs and the mixture was sonicated for 8 min (350 W). Then, free Rof was removed via low-speed centrifuge to obtain Rof@MVs (Rof, 80 μg/mL). 1.5 mL of Rof@MVs (Rof, 80 μg/mL) and 1.5 mL of 160 μg/mL MnO_2_ were homogenized by 4-min sonication (65 W). Then, these nanoparticles were processed for dynamic light scattering diameter analysis.

### Preparation of FITC-labelled MVs-based nanoparticles

MVs reacted with FITC at a mass ratio of 1:50, in NaHCO_3_–Na_2_CO_3_ buffer (0.15 mol/L, pH 9.0) for 12 h. Next, the product was dialyzed 4 times to remove free FITC and restored at − 80 °C. The FITC-labelled nanoparticles, including Rof@MVs-FITC, MnO_2_@MVs-FITC, Rof&MnO_2_@MVs-FITC, shared the same method with normal nanoparticles mentioned above.

### Determination of roflumilast in Rof@MVs

Rof@MVs were firstly dispersed in 3 times volume of acetonitrile for demulsification via ultrasound and then centrifuged to gather supernatant for HPLC analysis. The mobile phase was acetonitrile and Na_2_HPO_4_–NaH_2_PO_4_ buffer (0.01 mol/L, pH 4), with a volume ratio of 1:1, flowing at 1 mL/min. C18 column (4.6 × 250 mm, 5 μm, Agilent) served as the stationary phase and the signal was detected at 250 nm (Figure S3).

### Ex vivo simulation of MVs-based nanoparticles elimination

H_2_O_2_ was dropped into Rof @MVs (2 μg/mL Rof), MnO_2_@MVs (8 μg/mL MnO_2_) and Rof&MnO_2_@MVs (2 μg/mL Rof, 8 μg/mL MnO_2_) until the correspondent concentration. Then the nanoparticle solution was detected via ultraviolet–visible spectrophotometer (Figure S5).

### Stability of MVs-based nanoparticles in vitro

All nanoparticles were dispersed in simulated colon fluid (SCF, 0.05 mol/L KH_2_PO_4_, pH 7.4) at 37 °C and diameters were determined in various time intervals.

### Sustained release of roflumilast in vitro

1 mL Rof@MVs (40 μg/mL Rof) was dialyzed in 20 mL SCF (2% v/v Tween 80) at 37 °C for 24 h. At the indicated time, 1 mL SCF was aspirated for HPLC analysis and another 1 mL fresh SCF was complemented. Similarly, 1 mL Rof&MnO_2_@MVs (40 μg/mL Rof, 160 μg/mL MnO_2_) was used for the same measurement.

### Cell viability assay

RAW264.7 was seeded in 96-well plate at the density of 3 × 10^4^ per well for overnight. Then, cells were incubated with different concentration of nanoparticles for 24 h. Next, cell viability was examined with CCK-8 kit according to the manufacture protocol (Figure S6).

### Comparation of nanoparticle uptake between CT26 and RAW264.7

CT26 and Raw264.7 were seeded in 6-well plate at the density of 10^6^ per well for overnight, respectively. Then, cells were rinsed with 1× PBS and replenished with fresh medium, containing 1 μg/mL LPS. Next, Rof@MVs-FITC (Rof, 1.25 μg/mL), MnO_2_@MVs-FITC (MnO_2_, 5 μg/mL), Rof&MnO_2_@MVs-FITC (Rof, 1.25 μg/mL; MnO_2_, 5 μg/mL) and MVs-FITC (62.5 μg/mL) were added into the medium for 30-min incubation. After that, the medium was abandoned, and cells were rinsed three times and collected with 1× PBS for flow cytometry (BD FACS Callibur).

### Validation of the macrophage-target of MVs

Firstly, FITC-labelled MnO_2_ (MnO_2_-FITC) was obtained by the addition of 1 mg FITC into 3 mL of 160 μg/mL MnO_2_ nanoparticles in NaHCO_3_–Na_2_CO_3_ Buffer (0.015 mol/L, pH 9.0) for overnight reaction. Then, the product was dialyzed in deionized water for 5 times to eliminate the residual FITC. The synthesis of MnO_2_-FITC@MVs was same with that of MnO_2_@MVs as mentioned before. Next, RAW264.7 were grown in 6-well plate at the density of 10^6^ per well for overnight. Then, the medium was discarded, rinsed with 1× PBS and complemented with fresh medium, containing 1 μg/mL LPS. After that, RAW264.7 were incubated with MnO_2_-FITC (MnO_2_, 5 μg/mL), MnO_2_-FITC@MVs (MnO_2_, 5 μg/mL) and MVs (62.5 μg/mL) for 3 h. Lastly, cells were rinsed three times and collected with 1×  PBS for flow cytometry (BD FACS Callibur).

### Observation of nanoparticles in the macrophage

RAW264.7 was cultivated in the confocal dish at a density of 10^5^ per dish overnight. Then, cells were rinsed with 1× PBS and replenished with fresh medium, containing 1 μg/mL LPS. After that, cells were incubated with Rof@MVs-FITC (Rof, 1.25 μg/mL), MnO_2_@MVs-FITC (MnO_2_, 5 μg/mL), Rof&MnO_2_@MVs-FITC (Rof, 1.25 μg/mL; MnO_2_, 5 μg/mL) and MVs-FITC (62.5 μg/mL) for 0.5 h. Next, the medium was discarded, and cells were rinsed three times and observed in confocal laser scanning microscope (CLSM, Olympus FV3000). The excitation wavelength was 488 nm and the emission wavelength was 525 nm.

### The elimination of MnO_2_ in macrophage

RAW264.7 were seeded in 6-well plate at the density of 10^6^ per well for overnight. Then, cells were rinsed with 1× PBS and stimulated with LPS (1 μg/mL in fresh medium). Next, DCFH-DA (5 μmol/L), Rof@MVs (Rof, 1.25 μg/mL), MnO_2_@MVs (MnO_2_, 5 μg/mL), Rof&MnO_2_@MVs (Rof, 1.25 μg/mL; MnO_2_, 5 μg/mL) and MVs (62.5 μg/mL) were synchronously added into the medium for 30-min incubation. After that, the medium was abandoned, and cells were rinsed three times and collected with 1× PBS for flow cytometry (BD FACS Callibur).

### Influence of manganese in cytosolic cAMP

RAW264.7 were seeded in 96-well plate at the density of 1.5 × 10^4^ per well for overnight. Then, cells were rinsed with 1× PBS and stimulated with LPS (1 μg/mL in fresh medium). Next, various formulations of manganese were added into the medium for 30-min incubation, including high dose MnO_2_@MVs (MnO_2_@MVs-H, 5 μg/mL MnO_2_), low dose MnO_2_@MVs (MnO_2_@MVs-L, 0.5 μg/mL MnO_2_), MnO_2_ (5 μg/mL MnO_2_), MnCl_2_ (7.25 μg/mL) and MVs (62.5 μg/mL). After that, intracellular cAMP was detected by cAMP-Glo™ Assay in accordance with manufacture’s instruction.

### Modulation of nanoparticles in cytosolic cAMP

RAW264.7 were seeded in 96-well plate at the density of 1.5 × 10^4^ per well for overnight. Then, cells were rinsed with 1× PBS and stimulated with LPS (1 μg/mL in fresh medium). Next, various nanoparticles were added into the medium for 30-min incubation, including Rof@MVs (Rof, 1.25 μg/mL), MnO_2_@MVs (MnO_2_, 5 μg/mL), Rof&MnO_2_@MVs (Rof, 1.25 μg/mL; MnO_2_, 5 μg/mL) and MVs (62.5 μg/mL). After that, cellular cAMP was detected by cAMP-Glo™ Assay (Promega) in accordance with manufacture’s instruction.

### Quantification of TNF-α secreted from macrophage

RAW264.7 were seeded in 24-well plate at the density of 1.5 × 10^5^ per well for overnight. Then, cells were rinsed with 1× PBS and stimulated with LPS (1 μg/mL in fresh medium). Next, various nanoparticles were added into the medium for 6 h incubation, including Rof@MVs (Rof, 1.25 μg/mL), Rof@MVs (Rof, 2.5 μg/mL), MnO_2_@MVs (MnO_2_, 5 μg/mL), Rof&MnO_2_@MVs (Rof, 1.25 μg/mL; MnO_2_, 5 μg/mL), Rof&MnO_2_@MVs (Rof, 2.5 μg/mL; MnO_2_, 5 μg/mL) and MVs (62.5 μg/mL). After that, the supernatant was measured by TNF-α ELISA Kit (DAKEWE) in accordance with manufacture’s instruction.

### Protein analysis in Western Blot

RAW264.7 were seeded in 6-well plate at the density of 10^6^ per well for overnight. Then, cells were rinsed with 1× PBS and stimulated with LPS (1 μg/mL in fresh medium). Next, various nanoparticles were added into the medium for 30-min incubation, including Rof@MVs (Rof, 1.25 μg/mL), MnO_2_@MVs (MnO_2_, 5 μg/mL), Rof&MnO_2_@MVs (Rof, 1.25 μg/mL; MnO_2_, 5 μg/mL) and MVs (62.5 μg/mL). After that, cells were rinsed three times and harvested. Then, cells were dispersed in RIPA Lysis Buffer with proteinase inhibitors by sonication. The debris was removed by centrifugation at 14,000×*g*, 5 min at 4 °C. The supernatant was collected for SDS-PAGE (10%) electrophoresis. The protein was detected via p-CREB antibody and β-actin antibody.

### Establishment of DSS-induced colitis

All animal experiments were approved by Jinling Hospital (2021DZGKJDWLS-00143). Male C57BL/6 mice with 20–25 g, were purchased from Comparative Medicine Centre of Yangzhou University and housed in a 12 h light–dark cycle at 25 °C. Mice were raised 1 week for acclimation before random assignment. To construct murine colitis, mice were allocated randomly into groups and received 3% DSS in drinking water for 6 days. Healthy mice were supplied with ordinary drinking water during the experiment.

### In-vivo distribution of MVs-based nanoparticles

Male C57BL/6 mice with 20–25 g received 3% DSS for 6 days after 7-day acclimation. Then, mice were given enema with saline, Rof@MVs-FITC (Rof, 1 mg/kg), MnO_2_@MVs-FITC (MnO_2_, 4 mg/kg) and Rof&MnO_2_@MVs-FITC (Rof, 1 mg/kg and MnO_2_, 4 mg/kg). Various organs were gathered at the indicated time for fluorescent imaging via in vivo imaging system (IVIS, Berthold LB983 NC100).

### Prophylaxis of DSS-induced colitis

Before enema, mice were deprived of food for 12 h. Then, mice were anaesthetized with isoflurane and received enema on day 2, day 4 and day 6. The dose was Rof@MVs (Rof, 1 mg/kg), MnO_2_@MVs (MnO_2_, 4 mg/kg), Rof&MnO_2_@MVs (Rof, 1 mg/kg; MnO_2_, 4 mg/kg) and 5-ASA (1.25 mg/kg). Body mass was measured daily until sacrifice. On the day 9, mice were euthanized and colons were collected for length measurement.

### Pathology analysis of colon

The distal colon was collected and fixed in Carnot’s solution for 24 h for the sequent parafilm embedding. The morphology of colon tissue was demonstrated by hematoxylin and eosin (H&E) stain. Goblet cells in the mucus were manifested via Alcian blue stain.

### Detection of TNF-α in colon

The distal colon was collected and homogenized in the distal colon was collected (1 mmol/L PMSF). The debris was removed by centrifugation at 14,000×*g*, 5 min at 4 °C and the supernatant was collected. Then, the protein concentration was determined via BCA assay. After that, the supernatant was measured by TNF-α ELISA Kit (DAKEWE) in accordance with manufacture’s instruction.

### Detection of intestinal permeability

FITC-Dextran (average molecular weight 4kD, FD4) was used for the permeability examination. On the day 9, mice were deprived of food and water for 4 h and given FD4 (0.4 mg/g) via intragastric administration. Next, the blood was gathered retro-orbitally 3 h later. The fluorescent intensity of FD4 in serum was measured with microplate reader (excitation wavelength 488 nm, emission wavelength 525 nm).

### In vivo immunofluorescence imaging of Occludin

As tight junction protein was depleted in UC, Occludin was analyzed by immunofluorescence to evaluate the effect of nanoparticles. The distal colon was fixed in 4% paraldehyde for 24 h and embedded in OCT for frozen section. Sections were firstly blocked with 5% BSA for 30 min in room temperature. Then, the colon tissue was stained with rabbit Occludin polyclonal antibody (Proteintech, 1:2000) for overnight at 4 °C. Next, the primary antibody was washed away with PBST and sections were stained with CoraLite594–conjugated goat anti-rabbit IgG(H+L) for 2 h in room temperature. The tissue was rinsed three times with PBST to remove free antibody and stained with DAPI for 15–20 min. The fluorescent images were acquired via CLSM (Olympus FV3000).

### Macrophage-target of MVs-based nanoparticles in vivo

Male C57BL/6 mice with 20–25 g received 3% DSS for 6 days after 7-day acclimation. Then, mice were given enema with Rof&MnO_2_@MVs-FITC (Rof, 1 mg/kg and MnO_2_, 4 mg/kg). After 2 h, mice were sacrificed and the distal colons were collected and fixed in 4% paraldehyde for 24 h and embedded in OCT for frozen section. Sections were firstly blocked with 5% BSA for 30 min in room temperature. Then, the colon tissue was stained with rabbit F4/80 polyclonal antibody (Proteintech, 1:2000) for overnight at 4 °C. Next, the primary antibody was washed away with PBST and sections were stained with CoraLite594–conjugated goat anti-rabbit IgG(H+L) for 2 h in room temperature. The tissue was rinsed three times with PBST to remove free antibody and added with DAPI. The fluorescent images were acquired via CLSM (Olympus FV3000).

### Biosafety of MVs-based nanoparticles

Normal male C57BL/6 mice with 20–25 g, were housed and treated. Enema was performed on the day 2, 4 and 6 with the same dose in the experiment mentioned above, namely Rof@MVs (Rof, 1 mg/kg), MnO_2_@MVs (MnO_2_, 4 mg/kg), Rof&MnO_2_@MVs (Rof, 1 mg/kg; MnO_2_, 4 mg/kg) and 5-ASA (1.25 mg/kg). Everyday body mass weight was monitored and euthanasia was executed on day 9. Serum from each group was collected for further hepatocyte injury and renal toxicity examination. Additionally, heart, liver, spleen, colon, lungs and kidneys were obtained for H&E staining to evaluate organ injury.

### Microbiome analysis of colon

After mice were sacrificed, feces were collected for 16s rDNA sequencing in LC-Bio Technologies (Hangzhou) Co., Ltd. Briefly, total DNA was extracted with cetyltrimethylammonium bromide (CTAB). Then, the sequence was amplified in polymerase chain reaction (PCR). The PCR products were purified by AMPure XT beads (Beckman Coulter Genomics, Danvers, MA, USA) and quantified by Qubit (Invitrogen, USA). The amplicon pools were prepared for sequencing and the size and quantity of the amplicon library were assessed on Agilent 2100 Bioanalyzer (Agilent, USA) and with the Library Quantification Kit for Illumina (KapaBiosciences, Woburn, MA, USA), respectively. The libraries were sequenced on NovaSeq PE250 platform. The Shannon, Simpson, and Chao1 indices were calculated to assess the alpha diversity of each sample. PCA were used to assess beta diversity. The 30 most abundant communities at the genus level are shown by visualization methods, for example stack column.

### Statistical analysis

Graph Pad Prism 9.0 and Origin 9.0 were used for data statistics and statistical significance calculation. Data were presented as mean ± SD. Statistical analysis was performed via two-tail Student’s t test or one-way ANOVA multiple comparisons tests and Mann–Whitney test. *p < 0.05, **p < 0.01, ***p < 0.001 and ****p < 0.0001.

## Results and discussion

### Preparation and characterization of MVs-based nanoparticles

**Fig. 1:**
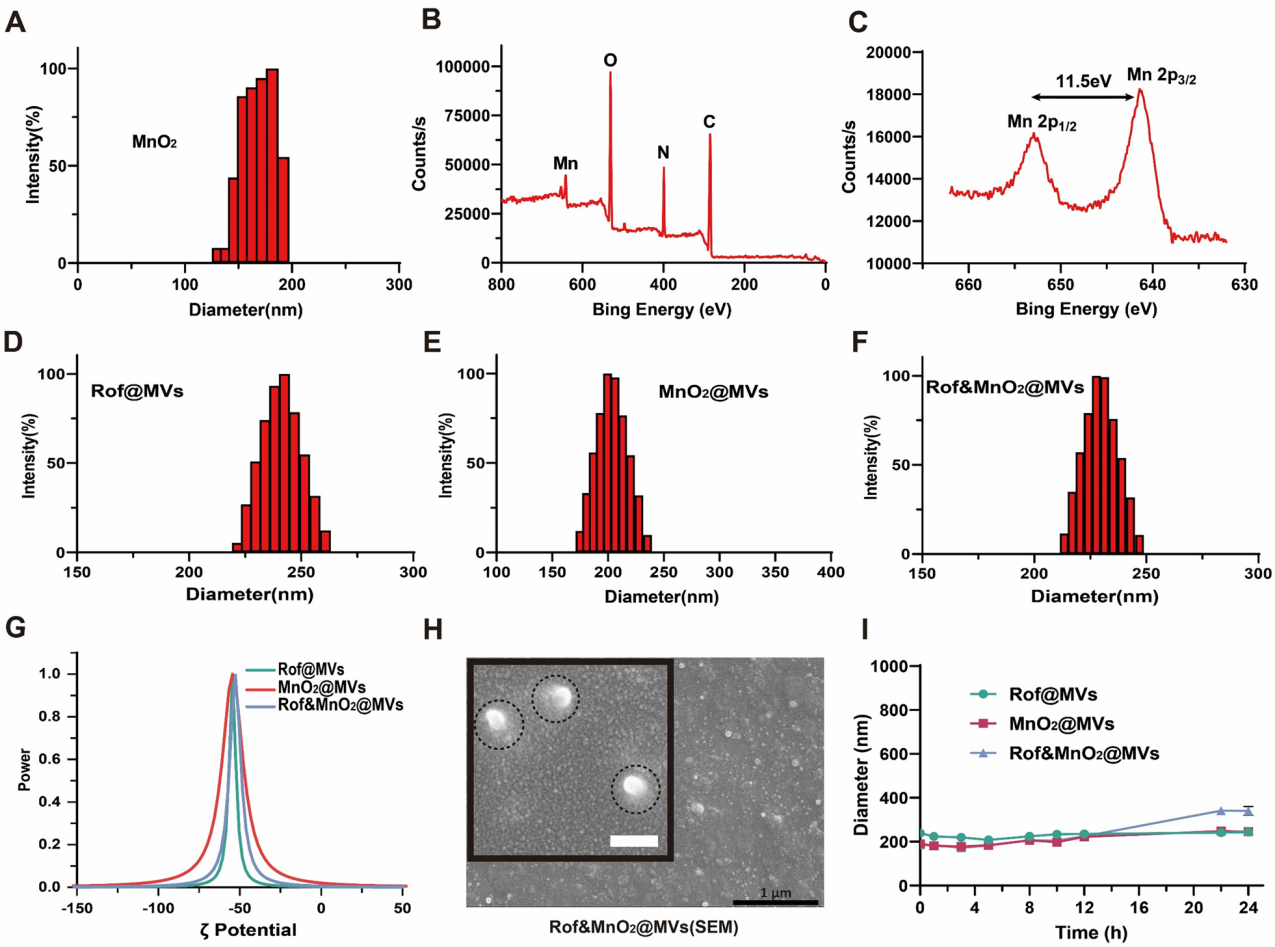
Characterization of MVs-based nanoparticles. **A** Dynamic light scattering diameter of MnO_2_. **B** X-ray photoelectron spectroscopy of MnO_2_. **C** The higher-resolution manganese spectra. **D** Dynamic light scattering diameter of Rof@MVs (Rof, 20 μg/mL). **E** Dynamic light scattering diameter of MnO_2_@MVs (MnO_2_, 80 μg/mL). **F** Dynamic light scattering diameter of Rof&MnO_2_@MVs (Rof, 20 μg/mL; MnO_2_, 80 μg/mL). **G** ζ potential of Rof@MVs (Rof, 20 μg/mL), MnO_2_@MVs (MnO_2_, 80 μg/mL) and Rof&MnO_2_@MVs (Rof, 20 μg/mL; MnO_2_, 80 μg/mL). **H** Scanning electron microscopy of Rof&MnO_2_@MVs (white scale bar 100 nm, black scale bar 1 μm). **I** Stability of MVs-based nanoparticles in simulated colon fluid (SCF, pH 7.2–7.8) at 37 °C

Probiotics EcN 1917 was firstly cultured in Luria–Bertani (LB) medium for 24 h before MVs collection. After that, MVs were collected by sonicating and ultracentrifuging. MnO_2_ nanoparticles were synthesized in two steps. Briefly, gelatin nanoparticles were obtained by the two step desolvation method and KMnO_4_ reacted with gelatin nanoparticles to prepare MnO_2_ nanoparticles. The diameters of both gelatin nanoparticles and MVs were detected via Dynamic light scattering (Figure S1). To synthesize Rof@MVs, roflumilast was encapsulated in MVs by repeated ultrasound. MnO_2_@MVs and Rof&MnO_2_@MVs were prepared in the similar way (for more preparation details, see “Experimental” section). The concentration of MnO_2_ nanoparticles and Rof in Rof @MVs, were quantified via formaldehyde oxime method and high-pressure liquid chromatography (HPLC), respectively (Figure S3–S4). The concentration of prepared MnO_2_ was about 1.3 mg/mL. Rof@MVs, with different Rof concentrations (40 μg/mL or 80 μg/mL), were prepared for further use. Dynamic light scattering (Fig. 1A) revealed the diameter of MnO_2_ (130–190 nm). The elemental mappings of MnO_2_ nanoparticles were depicted via the X-ray photoelectron spectroscopy (XPS). There were carbon, nitrogen, oxygen and manganese in MnO_2_ nanoparticles (Fig. 1B). Moreover, the higher-resolution manganese spectra manifested the existence of Mn 2p_1/2_ and Mn 2p_3/2_. The spin-orbital splitting distance between the Mn 2p_1/2_ and Mn 2p_3/2_ peaks, approximately 11.5 eV, verified the existence of MnO_2_ (Fig. 1C). Dynamic light scattering (DLS) displayed diameter distribution range of different nanoparticles, Rof@MVs (220–260 nm), MnO_2_@MVs (174–235 nm), Rof&MnO_2_@MVs (213–246 nm) (Fig. 1D–F). Another DLS diameter of Rof &MnO_2_@MVs (Rof, 40 μg/mL; MnO_2_, 80 μg/mL) was manifested in Figure S1C. Additionally, the ζ potential of these nanoparticles was about − 60 mV (Fig. 1G). Rof&MnO_2_@MVs exhibited a spherical morphology in the scanning electron microscopy (SEM, Fig. 1H). To verify the in-vivo stability, these nanoparticles were dispersed in simulated colon fluid (SCF, pH 7.2–7.8) at 37 °C for the dynamic diameter measurement within 24 h (Fig. 1I). It was found that there were insignificant fluctuations, which provided the opportunity for enema. Besides, in vitro sustained release profiles of Rof from both Rof@MVs and Rof&MnO_2_@MVs were determined via dialysis in the SCF and HPLC. The experimental results revealed that there was similar release behavior between Rof@MVs and Rof&MnO_2_@MVs (Figure S3B).

### Validation of MVs-based nanoparticles in targeting macrophage

As mentioned before, macrophage played an indispensable role in igniting inflammation in UC. Therefore, RAW 264.7 was selected as the model cell in the following experiments to testify the macrophage-target ability of nanoparticles in vitro.

**Fig. 2:**
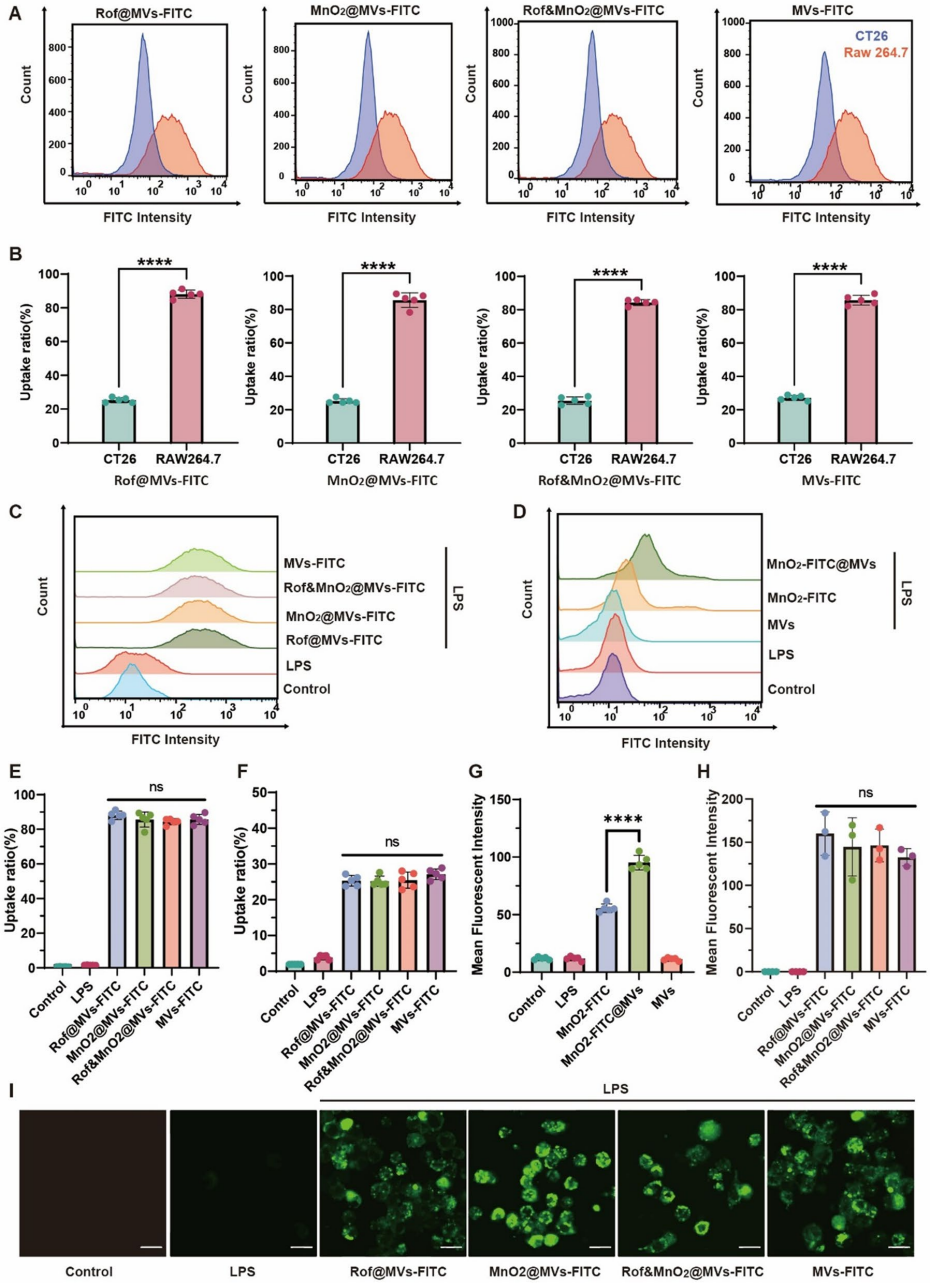
Macrophage-target of MVs-based nanoparticles. **A** Comparison of nanoparticle uptake in CT26 (blue) and RAW264.7 (orange) via flow cytometer. **B** Quantification of uptake ratio of the same nanoparticle between CT26 and RAW264.7 cells with signal intensity exceeding 100 were concluded (n = 5). **C** The uptake of various MVs-based nanoparticles in RAW264.7 via flow cytometer. **D** MVs could prompt the cargo MnO_2_ to accumulate in RAW264.7. **E** Quantification of uptake ratio of different nanoparticles in RAW264.7 (n = 5). **F** Quantification of uptake ratio of various nanoparticles in CT26 (n = 5). **G** Analysis of MnO_2_ uptake in RAW264.7 with or without MVs (n = 5). **H** Mean fluorescent intensity of FITC-labelled nanoparticles in RAW264.7 when observed under CLSM. (n = 3). **I** Representative fluorescent graph of FITC-labelled nanoparticles in RAW264.7 (scale bar 20 μm). These data were manifested as mean ± SD. *p < 0.05, **p < 0.01, ***p < 0.001, ****p < 0.0001, ns (none significance). Data was statistically analyzed via two-tail Student’s t test or one-way ANOVA multiple comparisons tests (Tukey’s test was used for comparison of multiple groups)

Firstly, the cell viability of various nanoparticles was detected via CCK-8 kit (Figure S6). The concentrations, which were less than IC_50_, were selected for further experiment. Due to the inherent PAMP, it was deduced that MVs tended to accumulate within macrophage. Fluorescein isothiocyanate (FITC)-labelled MVs (MVs-FITC) were fabricated as both vector and tracker. Besides, CT26 was selected as a contrast to simulate colon epithelial cells, in order to explore cellular target of MVs-based nanoparticles in vitro. When incubated with FITC-labeled miscellaneous nanoparticles in 0.5 h, both CT26 and RAW264.7 were collected for cytometry analysis (Fig. 2A). Cells, whose fluorescent intensity exceeded 100, were selected for uptake ratio analysis. The uptake ratio indicated that macrophage was conspicuously prone to engulf these nanoparticles in comparison with CT26 (Fig. 2B). MVs were the potential shuttle for the precise delivery into macrophage. It was found that in the same cell, there was inconspicuous uptake difference among various MVs-based nanoparticles (Fig. 2C, E, F). For a more visual embodiment, macrophage was treated with Rof@MVs-FITC, MnO_2_@MVs-FITC, Rof&MnO_2_@MVs-FITC and MVs-FITC and then analyzed via confocal laser scanning microscope (CLSM). It was found that there was insignificant phagocytosis difference among these nanoparticles (Fig. 2H). These graphs reflected a preeminent uptake capacity of macrophage (Fig. 2I). To verify the hypothesis that MVs propelled the uptake of cargo in macrophage, MnO_2_ was tagged with FITC and entrapped in MVs for a further examination. The outcome reflected that MnO_2_-FITC@MVs were more inclined towards accumulating in macrophage, in comparation with MnO_2_-FITC (Fig. 2D, G). This furtherly confirmed the speculation that MVs indeed rendered MnO_2_ the capability of targeting macrophage. Taken together, MVs-based nanoparticles could specifically accumulate in macrophage, owing to the biomimick trait.

### Synergistical influence of roflumilast and MnO_2_ on cAMP concentration

**Fig. 3:**
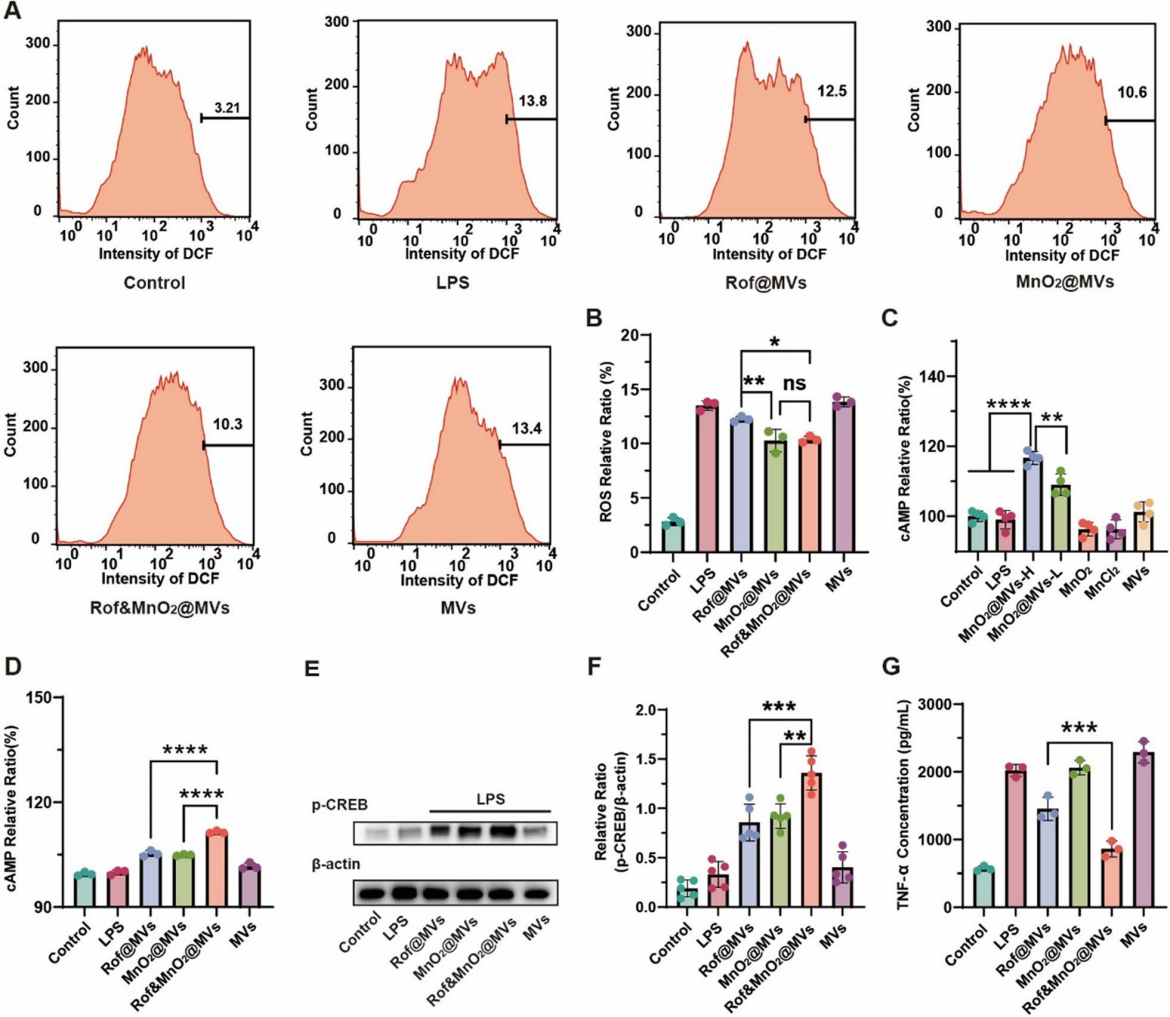
The underlying biological mechanism of roflumilast and MnO_2_. **A** The intracellular decomposition of MnO_2_ by H_2_O_2_, illustrated via flow cytometer. **B** Quantifying ROS relative ratio of different nanoparticles in RAW264.7. Cells with signal intensity surpassing 1000 were analyzed (n = 3). **C** The effect of diverse formulations of Mn on the cytosolic cAMP. MnO_2_@MVs-H means high concentration (5 μg/mL MnO_2_). Similarly, MnO_2_@MVs-L represents low concentration (0.5 μg/mL MnO_2_). The Mn concentration of both MnO_2_ and MnCl_2_ was 5 μg/mL. To obtain the relative ratio, the concentrations of cAMP in groups were divided by the control (n = 4). **D** The effect of MVs-based nanoparticles on the cytosolic cAMP. (n = 3). **E** The phosphorylation of CREB in various MVs-based nanoparticles in Western Blot. **F** The relative quantification of p-CREB in Western Blot (n = 5). **G** The synergistical efficacy of roflumilast and MnO_2_ in inhibiting TNF-α secretion from RAW264.7 (n = 3). Rof@MVs (Rof, 1.25 μg/mL), MnO_2_@MVs (MnO_2_, 5 μg/mL), Rof&MnO_2_@MVs (Rof, 1.25 μg/mL; MnO_2_, 5 μg/mL) and MVs (62.5 μg/mL). The concentration ratio of roflumilast and MnO_2_ is 1:4. These data were manifested as mean ± SD. *p < 0.05, **p < 0.01, ***p < 0.001, ****p < 0.0001, ns (none significance). Data was statistically analyzed via one-way ANOVA multiple comparisons tests (Tukey’s test was used for comparison of multiple groups)

Since MnO_2_ reacted with intracellular H_2_O_2_ to generate Mn^2+^, reactive oxygen species (ROS) detection was performed with probe 2′,7′-dichlorofluorescein diacetate (DCFH-DA) to directly confirm the process [34]. Stimulated macrophage (LPS, 1 μg/mL) was incubated with diverse categories of nanoparticles and DCFHA for flow cytometry. Cells, of which fluorescent intensity transcended 1000, were selected for relative ratio analysis. There was less proportion of 2′,7′-dichlorofluorescein (DCF) fluorescent intensity in MnO_2_@MVs and Rof&MnO_2_@MVs than other nanoparticles (Fig. 3A, B).

Since Mn^2+^ is reputed as a cofactor of AC, it is considered that MnO_2_ possesses the similar property as a precursor. Firstly, macrophage was activated with lipopolysaccharide (LPS, 1 μg/mL), and various formulations of Mn were investigated for the positive regulation on cAMP production, including MnO_2_@MVs, MnO_2_ and MnCl_2_. The concentrations of cAMP in all groups, were divided by the control group to obtain cAMP relative ratio. The experimental results displayed that MnO_2_@MVs could promote cAMP production in a dose-independent effect (Fig. 3C). Otherwise, there was no significant discrimination between MnO_2_ and MnCl_2_ in the same concentration of Mn, which indirectly indicated the advantage of MVs. The phenomenon that MnCl_2_ had no influence on cAMP, ascribed to short incubation time and different transportation pathway, in reference with previous studies. Next, it was explored whether codelivery of roflumilast and MnO_2_ could generate more cAMP. Macrophage was cultured with series of MVs-based nanoparticles in 0.5 h to determine the cytosolic cAMP. The results uncovered that Rof@MVs, MnO_2_@MVs, and Rof&MnO_2_@MVs certainly increased cAMP in macrophage while MVs not (Fig. 3D). Now that cAMP combined with PKA to phosphorylate the downstream protein CREB to implement anti-inflammation functions, Western Blot (WB) was selected to depict this process. It was manifested that there was more p-CREB in Rof&MnO_2_@MVs than any other groups (Fig. 3E, F).

To explore inhibitory effect in TNF-α secretion from macrophage, RAW 264.7 were stimulated with LPS (1 μg/mL) then cultured with different nanoparticles, and the supernatant was collected for enzyme linked immunosorbent assay (ELISA). Different concentration ratios of roflumilast and MnO_2_ were also explored. Rof&MnO_2_@MVs could superiorly impede the TNF-α secretion from macrophage, compared with Rof@MVs, (Fig. 3G) when the concentration ratio of roflumilast and MnO_2_ is 1:4. However, when the concentration ratio of roflumilast and MnO_2_ is 1:2, there was insignificant difference between Rof@MVs and Rof&MnO_2_@MVs (Figure S7). In addition, MnO_2_@MVs manifested no inhibitory effect in that Mn^2+^ might also conjugate with others proteins in inflammatory pathway which counteracted cAMP-associated anti-inflammatory efficacy (Fig. 3G). Conclusively, more cAMP was produced in the existence of both roflumilast and MnO_2_, in comparison with sole roflumilast.

### Biodistribution of various MVs-based nanoparticles

**Fig. 4:**
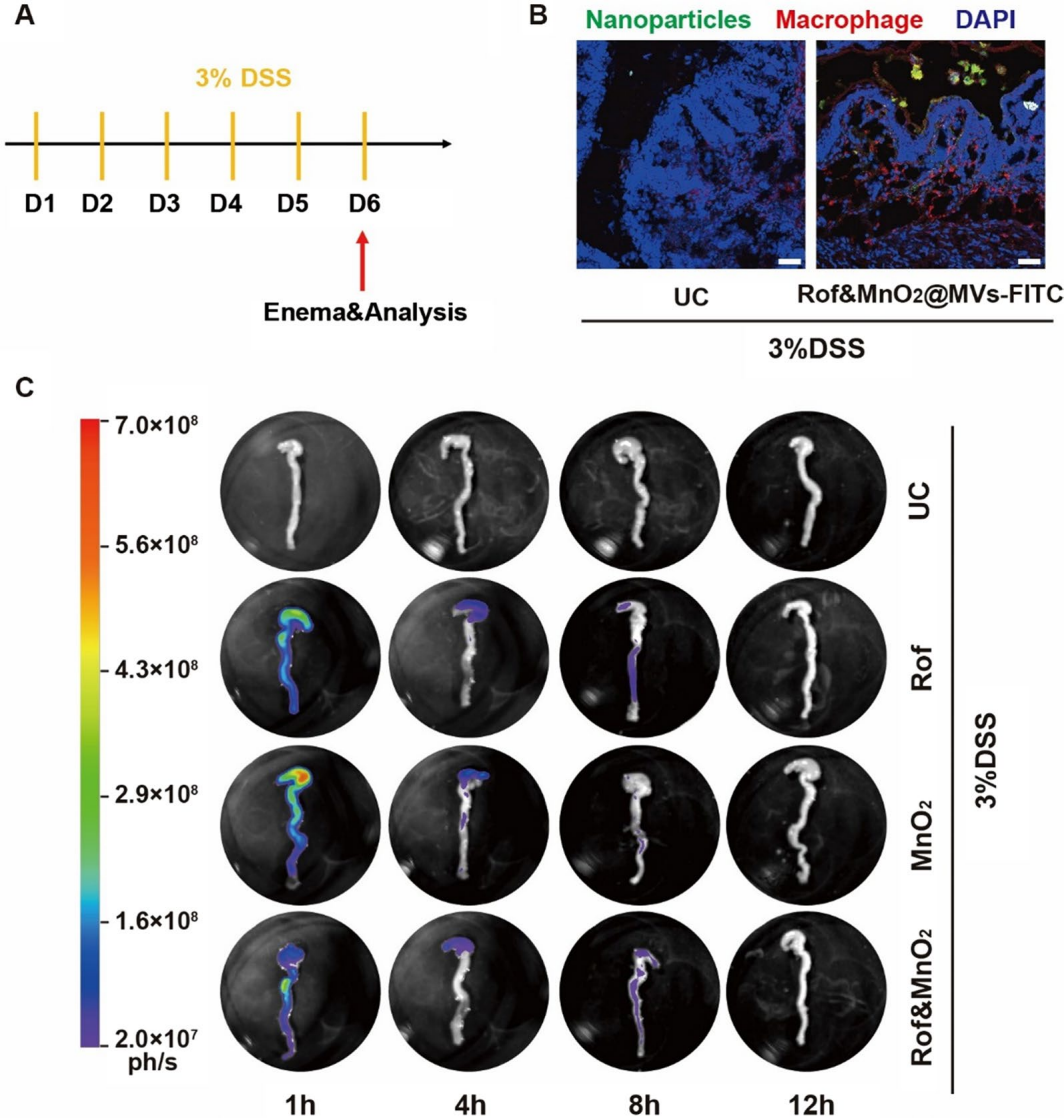
The biodistribution of nanoparticles in colon after enema. **A** Experiment scheme. 3% (wt %) DSS was used to establish colitis via free drinking from day 1 to day 6. Mice were given enema with FITC-labelled nanoparticles and analyzed on day 6. **B** Representative fluorescent imaging of nanoparticles uptake in macrophage in Vivo (scale bar 20 μm). **C** The detention time of FITC-labelled nanoparticles in colon after enema via in vivo imaging system. Abbreviations: Rof, Rof@MVs-FITC; MnO_2_, MnO_2_@ MVs-FITC; Rof&MnO_2_, Rof&MnO_2_@ MVs-FITC

Considering that macrophage was activated and engulfed bacterial from lumen in colitis, it was deemed that MVs-based nanoparticles were macrophage-target in the colon due to preeminent biomimick. To verify this, FITC-labeled MVs were designed to deliver roflumilast and MnO_2_ simultaneously. After enema, colons were collected for immunofluorescent assay and it was found that macrophage swallowed nanoparticles indeed (Fig. 4B). This validated the macrophage-target of MVs-based nanoparticles histologically.

To explore the accumulation of nanoparticles in different organs, MVs-FITC served as the vehicle for delivering Rof or MnO_2_. Mice with colitis were given enema with Rof@MVs-FITC, MnO_2_@MVs-FITC and Rof&MnO_2_@MVs-FITC. Then, organs were collected after euthanasia at the indicated time (1 h, 4 h, 8 h and 12 h) for imaging by in vivo imaging system. It was found that these nanoparticles were excreted after 12 h in the colon (Fig. 4C). Additionally, there was insignificant fluorescence in other organs including liver, heart, spleen, lung and kidney (Figure S8), implying that these nanoparticles mainly remained in the colon rather than others.

### Amelioration of DSS-induced colitis in mice by nanoparticles

**Fig. 5:**
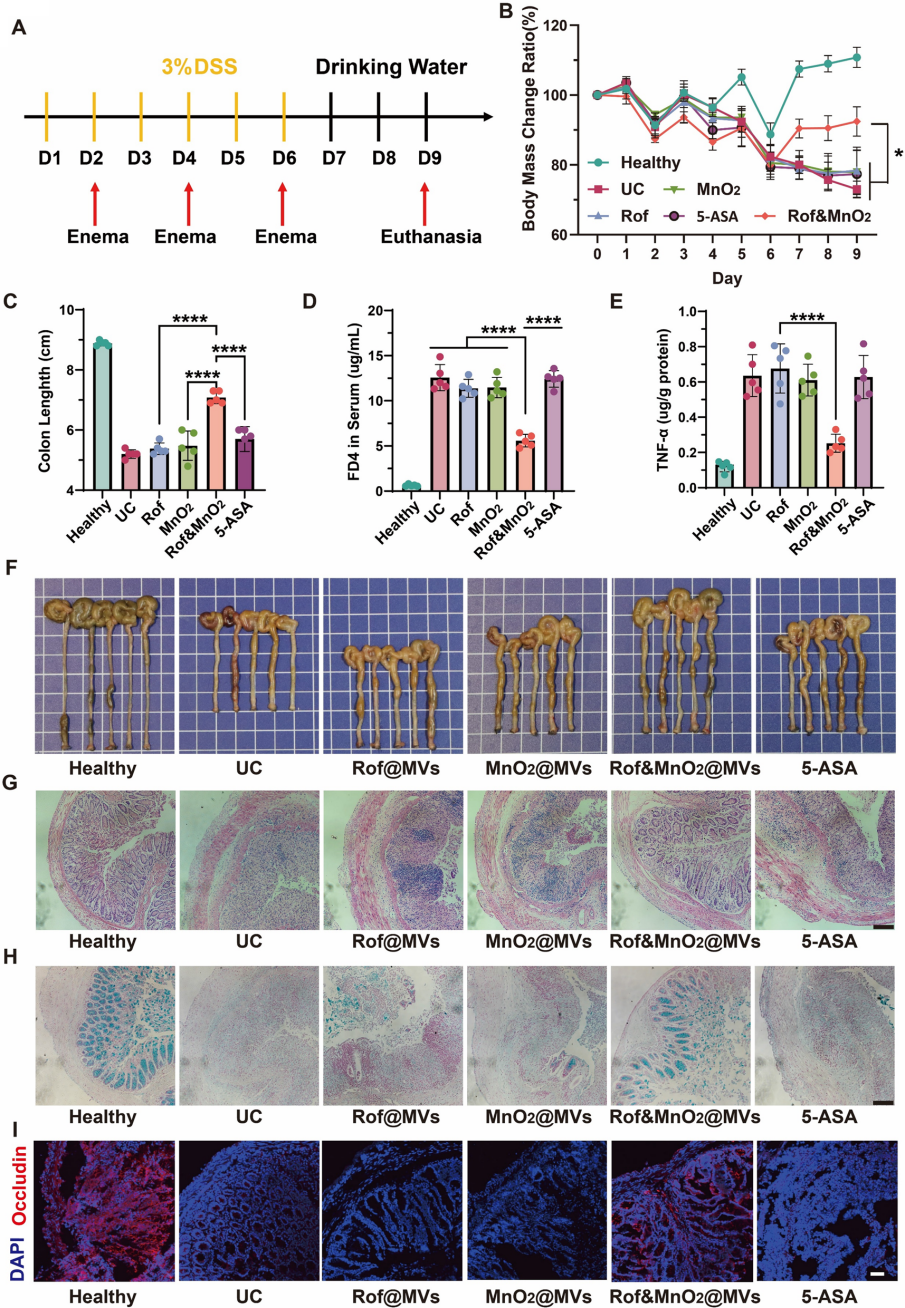
The efficacy of MVs-based nanoparticles in ameliorating DSS-induced colitis. **A** The scheme of experiment. Mice were firstly raised 7 days for acclimation before colitis establishment. Then, 3% (wt %) DSS was used to establish colitis via free drinking from day 1 to day 6. During this process, enema was performed on day 2, 4 and 6 with Rof (Rof@MVs, roflumilast 1 mg/kg), MnO_2_ (MnO_2_@MVs MnO_2_, 4 mg/kg), Rof&MnO_2_ (Rof&MnO_2_@MVs, roflumilast, 1 mg/kg and MnO_2_, 4 mg/kg) and 5-ASA (1.25 mg/kg). From day 7, DSS was substituted with drinking water until euthanasia. On day 9, mice were sacrificed and colon was collected for further analysis. **B** Dynamic body weight mass in different groups during the experiment (n = 5). **C** Measurement of colon length in various groups (n = 5). **D** Analysis of intestinal permeability with FD4 (n = 5). **E** TNF-α level in colon tissue (n = 5). **F** Photograph of colon from mice (n = 5). The grid behind the specimen is 1 cm × 1 cm. **G** Representative images of H&E stain of colon in each group (scale bar 100 μm). **H** Representative graphs of goblet cells in each group by Alcian blue staining (scale bar 100 μm). **I** Immunofluorescence examination in Occludin of colon from each group (scale bar 20 μm). These data were manifested as mean ± SD. *p < 0.05, **p < 0.01, ***p < 0.001, ****p < 0.0001. Data was statistically analyzed via two-way or one-way ANOVA multiple comparisons tests (Tukey’s test was used for comparison of multiple groups)

It was estimated whether MVs-based nanoparticles could alleviate clinical manifestations of colitis in a murine model. For in-vivo investigation, C57BL/6 mice were selected and distributed randomly into 6 groups, including health, UC, Rof@MVs, MnO_2_@MVs, Rof&MnO_2_@MVs and 5-ASA. To explore whether Rof&MnO_2_@MVs had superior anti-inflammatory efficacy than 5-ASA, the dose of 5-ASA is slightly higher than that of Rof. The experiment scheme was illustrated in the Fig. 5A. Based on the biodistribution experiment and circumventing the accidental death risk in the process, rectal administration was performed after 12-h fasting every 2 days.

To smoothly perform enema, all mice were deprived of food for 12 h before enema. As fasting could negatively affected body weight, body weight in all groups decreased on the day 2, 4 and 6. When the scheduled enema was finished, body weight in the healthy bounced to the normal level. Besides, it was indicated that Rof&MnO_2_@MVs could mitigate body weight loss while others not (Fig. 5B). This revealed that Rof&MnO_2_@MVs did alleviate clinical manifestations. Moreover, 4 kDa FITC-dextran (FD4) was orally gavaged to examine the intestinal permeability on the day 9. The concentration of FD4 in the serum was less in Rof&MnO_2_@MVs, compared with others (Fig. 5D). These experimental evident confirmed that Rof&MnO_2_@MVs could maintain the mucosa permeability in some degree. In addition, Rof&MnO_2_@MVs also obviously prevented colon from shrinking compared with other groups (Fig. 5C, F). Next, colon tissue was collected for TNF-α determination. It was found that there was less TNF-α in colon tissue from group Rof&MnO_2_@MVs (Fig. 5E). Less tissue damage existed in Rof&MnO_2_@MVs (Fig. 5G). Besides, more goblet cells were maintained in Rof&MnO_2_@MVs, implying that codelivery of roflumilast and MnO_2_ could exert a protective effect on mucosa (Fig. 5H). As tight-conjunction protein was depleted in colitis [35], Occludin was chosen as the representative biomarker to evaluate the therapeutic efficacy of MVs-based nanoparticles and 5-ASA. It was demonstrated that except for healthy mice, there was more Occludin in Rof&MnO_2_@MVs than other groups in immunofluorescent assay (Fig. 5I). These results indicated that Rof&MnO_2_@MVs possessed superior efficacy than other nanoparticles. Additionally, Rof&MnO_2_@MVs indeed improved manifestation of UC mice, while 5-ASA not. It was speculated that the dose of 5-ASA was insufficient to exert any anti-inflammation efficacy.

To explore the biocompatibility, normal mice were housed and treated, similar with the prophylaxis (Figure S10A). Dynamic body mass weight remained relatively stable after enema, which implied that these nanoparticles were unable to engender body weight loss (Figure S10B). The H&E sections of various organs revealed insignificant tissue injury (Figure S11). Besides, there was no difference in the ALT (alanine transaminase), AST (aspartate transaminase), BUN (blood urea nitrogen), CREA (creatinine) in comparation with control group, indicating that these MVs-based nanoparticles rarely caused hepatocyte injury and renal dysfunction (Figure S10C-F).

In conclusion, codelivery of roflumilast and MnO_2_ could obstruct the aggravation of UC in murine model, due to the macrophage-target of MVs.

### Modulation of gut microbiome in colitis mice by nanoparticles

**Fig. 6:**
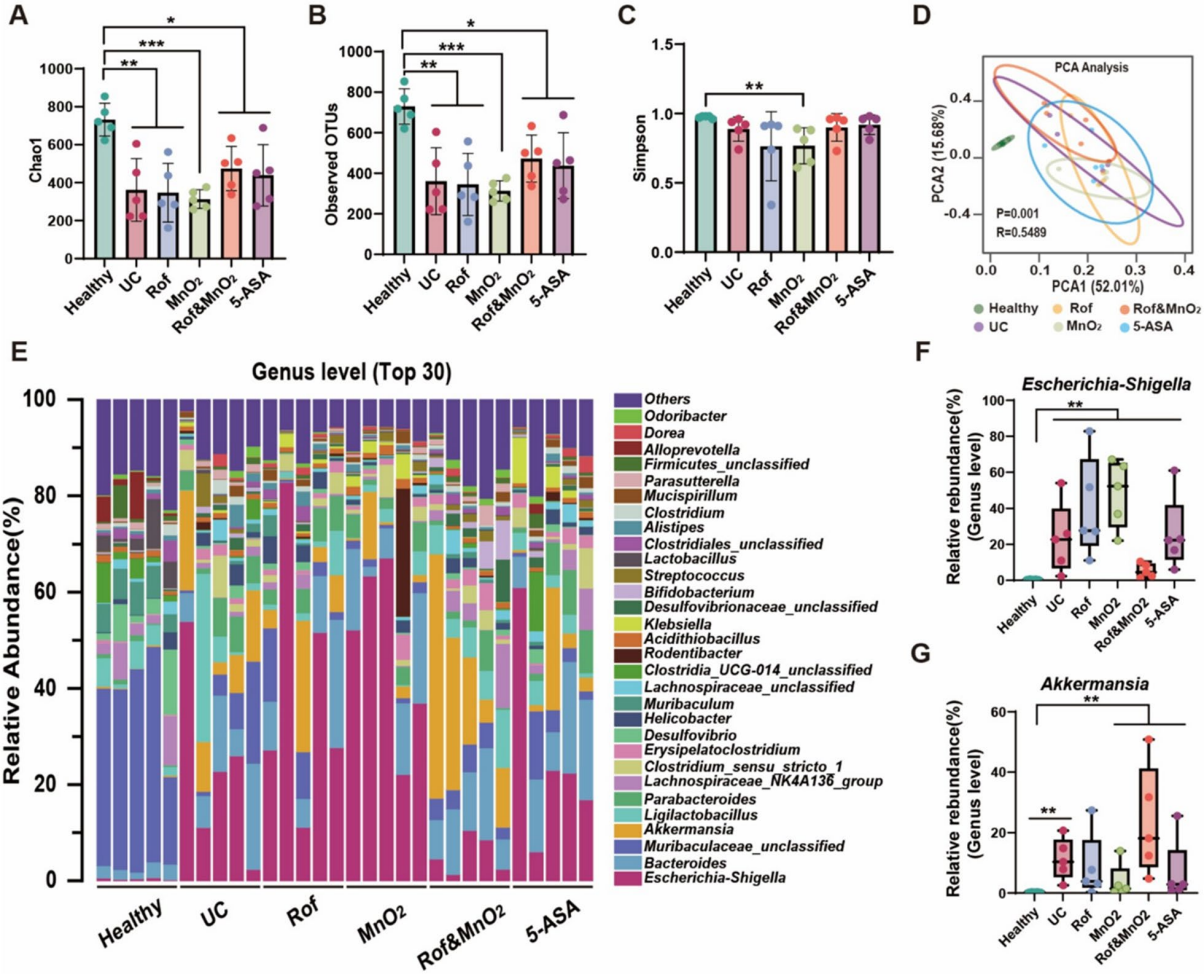
The efficacy of MVs-based nanoparticles in gut microbes. **A** The Chao1 of different groups (n = 5). These data were manifested as mean ± SD. **B** The observed operation taxonomy units (OTUs) of different groups (n = 5). These data were manifested as mean ± SD. **C** The Simpson diversity index of various groups (n = 5). These data were manifested as mean ± SD. **D** Principal components analysis (PCA) of intestinal microorganism. **E** Representative top 30 bacteria in genus level. **F** The relative abundance of *Escherichia–Shigella* in genus (n = 5). **G** The relative abundance of *Akkermansia* in genus (n = 5). Rof: Rof@MVs; MnO_2_: MnO_2_@MVs; Rof&MnO_2_: Rof&MnO_2_@MVs. *p < 0.05, **p < 0.01, ***p < 0.001, ****p < 0.0001. Data in **A**, **B** was statistically analyzed via one-way ANOVA multiple comparisons tests (Tukey’s test was used for comparison of multiple groups). Data in **C**, **F**, **G** was statistically analyzed via nonparametric tests (Mann–Whitney test was used for comparison of two groups)

As there exists the dysregulation on microbiome in UC, it has been recognized that the mitigating diversity and altering composition of microbiota are cardinal traits [36]. Thus, the faeces of colitis mice were collected to detect the component of the gut microbiota by 16S ribosomal DNA (rDNA) gene sequencing of the V3–V4 regions. In comparation with healthy mice, other groups manifested less Chao1 (Fig. 6A) and observed OTUs (Fig. 6B), which represented lower bacteria abundance. This perhaps ascribed to inflammation reaction in colon. Additionally, MnO_2_@MVs decreased Simpson diversity index while others not (Fig. 6C). In β diversity, the principal components analysis (PCA) demonstrated microbe community structures in the healthy group was distinctively different from the remnant groups (Fig. 6D). Others illustrated insignificant difference, owing to colon severe (Rof@MVs, MnO_2_@MVs and 5-ASA) or mild inflammation (Rof&MnO_2_@MVs). In addition, MVs-based nanoparticles were able to modulate the abundance ratio of probiotics (for example, *Akkermansia*) and pathogenic microorganism (*Escherichia–Shigella*) in colon [37]. Top 30 abundant bacteria were summarized in genus level (Fig. 6E). It was found that the relative abundance of pathogenic *Escherichia–Shigella* was less in Rof&MnO_2_@MVs than other treatment groups (Fig. 6F). Simultaneously, there was more proportion of probiotics *Akkermansia* in Rof&MnO_2_@MVs (Fig. 6G). Top 5 bacteria from different groups in genus level also found more *Akkermansia* and less *Escherichia–Shigella* in Rof&MnO_2_@MVs (Figure S9). These consequences indicated these nanoparticles could regulate gut microbiome to ameliorate DSS-induced colitis in some degree.

It has been widely accepted that macrophage plays an important role in the occurrence and development of ulcerative colitis. Regulating the physiological activity of macrophage has been regarded as an effective way to lessen the inflammation level. Since high level of cAMP can effectively inhibit pro-inflammation cytokine production in macrophage, Rof can become the candidate for UC therapy. However, Rof is hydrophobic and lack of cell target. Hitherto, there has been few works involved in synthesizing nanoparticles for Rof target delivery. Enlighted from the process that macrophage engulfs bacteria, it can be speculated that MVs, from probiotics, is a natural drug delivery platform with good biocompatibility for targeting macrophage. In this research, MVs-based nanoparticles were prepared to tackle the intrinsic drawbacks of Rof. Both Rof and MnO_2_ were encapsulated in MVs for macrophage-target delivery. MVs facilitated the Rof hydrophilicity and released Rof in a sustained way. In macrophage, Rof&MnO_2_@MVs exerted a “two‑birds‑one‑stone” effect to elevate cytosolic cAMP concentration. One the one hand, Rof deactivated PDE4 and inhibited the degradation of cAMP. Besides, MnO_2_ could be transferred into Mn^2+^ in macrophage. Then, Mn^2+^ could bind with adenylate cyclase and improve the catalytical capacity to produce more cAMP from ATP. More cytosolic cAMP could exert stronger anti-inflammation effect, which indicated that less Rof still maintained the same efficacy and less adverse reactions would occur. Although many small molecular drugs have been utilized in UC management (mesalamine, glucocorticoid and immunosuppressants), some patients appeared less responsive to them during the process [38–41]. Thus, developing new drugs is urgent. It is anticipated that Rof&MnO_2_@MVs can be introduced into the UC management in the future.

## Conclusion

Previous researches have uncovered that roflumilast can downregulate TNF-α synthesis via increasing cAMP in macrophage, but the intrinsic defect mentioned before still restrain its usage in UC. Since it has been validated that Mn^2+^ can propel cAMP production, it is proposed that roflumilast and Mn^2+^ can synergistically produce more cAMP in macrophage to ameliorate UC. Therefore, we designed MVs-based nanoparticles to co-deliver roflumilast and MnO_2_ into macrophage. These nanoparticles manifested excellent macrophage-target both in vitro and in vivo, which rendered the cargo accurate modulation of macrophage. Additionally, co-delivery of roflumilast and MnO_2_ produced more cAMP and less TNF-α in macrophage when compared with roflumilast. Moreover, in contrast to other nanoparticles and 5-ASA, Rof&MnO_2_@MVs mitigated UC manifestation in mice model such as impeding weight loss, protecting goblet cells and improving intestinal mucosa barrier. Besides, Rof&MnO_2_@MVs could regulate colon microbe including prompting probiotic and alleviating pathogenic bacteria. Although roflumilast (Daliresp®) has been under clinic trial in UC, some side effects are still inevitable due to lack of target. Since manganese is one indispensable trace element, this codelivery approach is potential in further clinical application for modulating precisely macrophage. These nanoparticles could serve as candidates for alleviating inflammation in acute colitis. However, rectal administration may restrict the utility in chronic colitis, due to the narrow lumen. Thus, further investigations on new oral formulations should be considered. Conclusively, one probiotic-based nanoparticle for precisely modulating cAMP in macrophage was prepared and it can be a latent candidate for UC treatment in the future.

### Supplementary Information

Supplementary Material 1.

## Data Availability

All data needed to support the conclusions are present in the paper and/or the supporting information. Additional data related to this paper may be requested from the authors.
