# Supplementary material for: MnO2 and roflumilast-loaded probiotic membrane vesicles mitigate experimental colitis by synergistically augmenting cAMP in macrophage
Source: J Nanobiotechnology. 2024 May 28;22:294. doi: 10.1186/s12951-024-02558-6 (PMC11131305; doi:10.1186/s12951-024-02558-6)
Supplement: Supplementary file 1 — Supplementary Material 1. [file 12951_2024_2558_MOESM1_ESM.docx]

Supporting Information for

**MnO_2_ and roflumilast-loaded probiotic** **membrane vesicles mitigate experimental colitis by synergistically augmenting cAMP in macrophage**

Chengjun Song^1^, Jiamin Wu^1^, Jinhui Wu^1,3,4^, Fangyu Wang ^1,2^*

^1^State Key Laboratory of Pharmaceutical Biotechnology, Medical School, Nanjing University, Nanjing 210093, China.

^2^Department of Gastroenterology and Hepatology, Jinling Hospital, Affiliated Hospital of Medical School, Nanjing University, Nanjing 210002, China.

^3^Institution of Drug R&D, Nanjing University, Nanjing 210093, China.

^4^Chemistry and Biomedicine Innovation Center, Nanjing University, Nanjing 210093, China.

*Corresponding authors.

**Email**: [wangfy65@nju.edu.cn](mailto:wangfy65@nju.edu.cn) (Fangyu Wang).


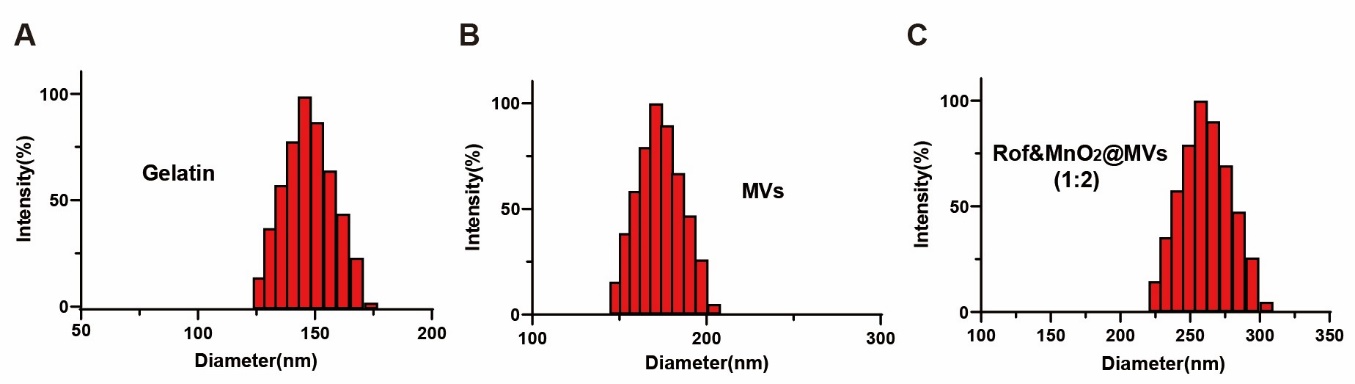


**Figure S1**. Characterization of gelatin nanoparticles and MVs. (A) Dynamic light scattering diameter of gelatin nanoparticles. (B) Dynamic light scattering diameter of MVs. (C) Dynamic light scattering diameter of Rof&MnO_2_@MVs with concentration ratio of 1:2. (Rof, 40 μg/mL; MnO_2_, 80 μg/mL).

**
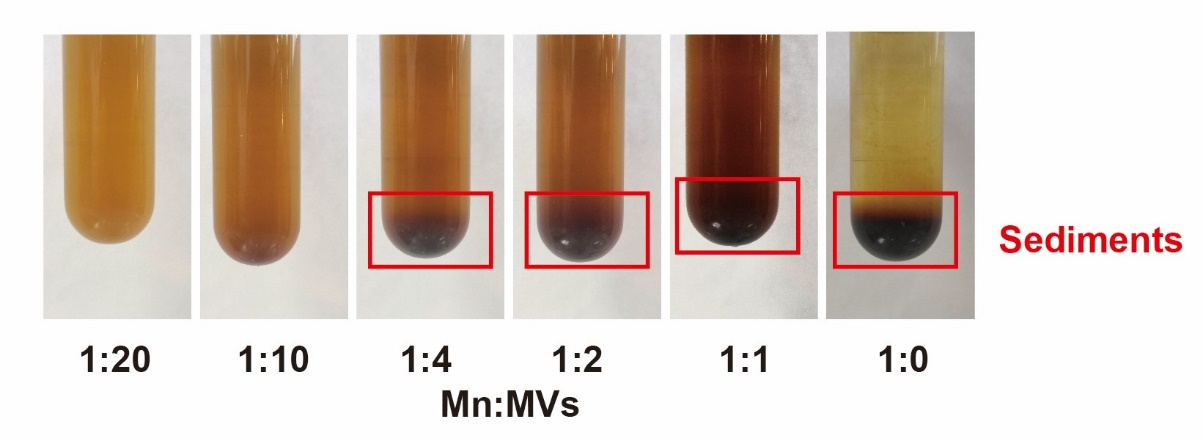
Figure S2**. Investigation on different ratio of MnO_2_ and Rof@MVs. Rof&MnO_2_@MVs were prepared in various ratio of MnO_2_ and Rof@MVs. Then Rof&MnO_2_@MVs were frozen and thawed from -20℃. Mn: MVs means the proportion of manganese concentration in MnO_2_ and protein concentration of MVs, respectively.


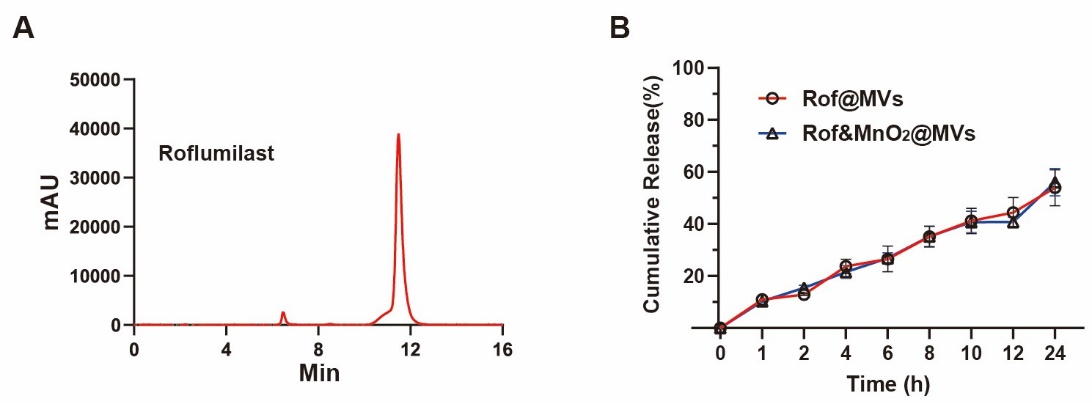


Figure S3. HPLC analysis of roflumilast (Rof) and Rof@MVs. (A) roflumilast (50 μg/mL). (B) Sustained release profiles of roflumilast from nanoparticles in SCF*.*


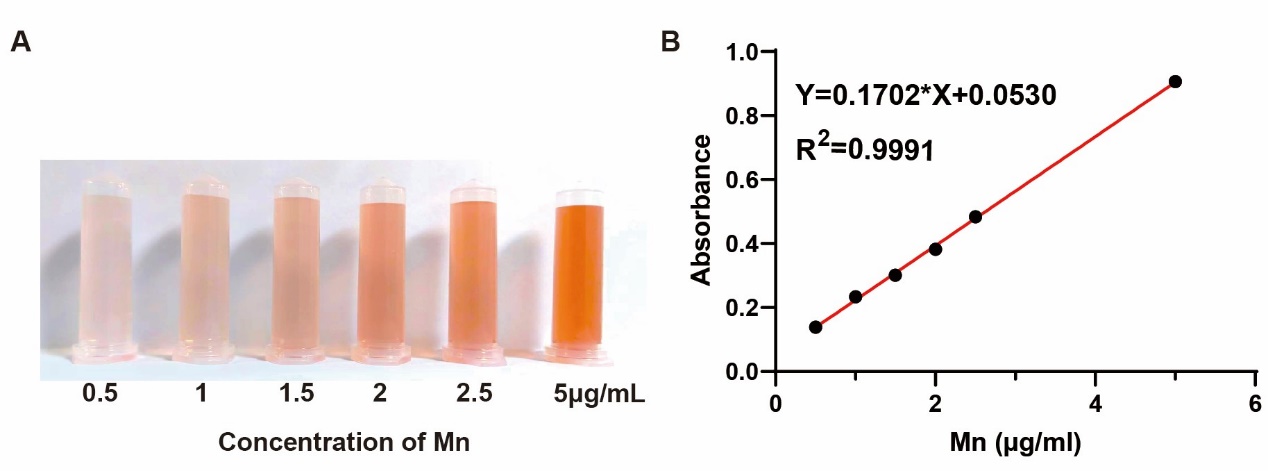


**Figure S4.** Quantification of Mn^2+^ in formaldehyde oxime method. (A) Different concentrations of Mn^2+^. (B) Standard curve of Mn^2+^.


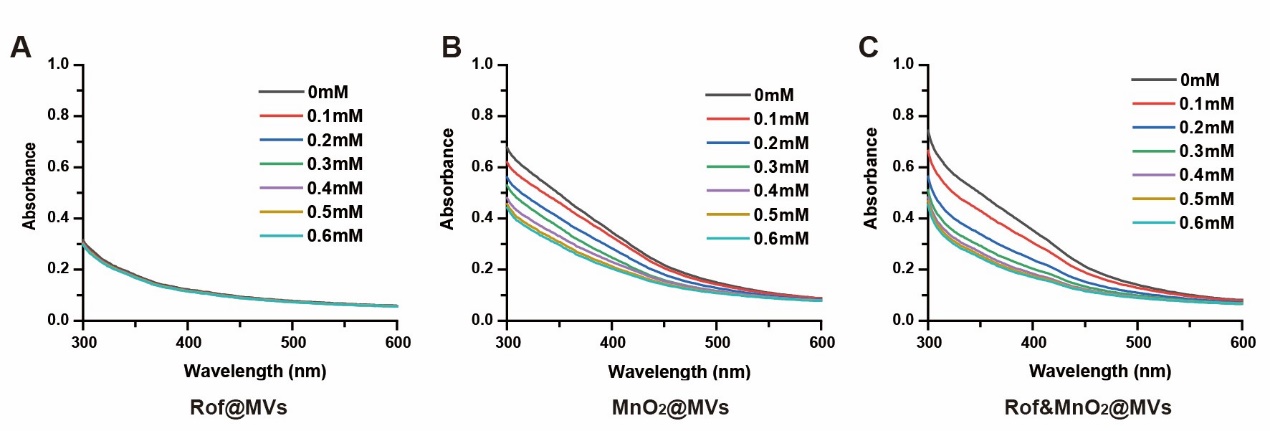


**Figure S5.** Ultraviolet-visible spectrum of different nanoparticles in H_2_O_2_ solution with various concentrations. (A) Rof@MVs. (B) MnO_2_@MVs. (C) Rof&MnO_2_@MVs.


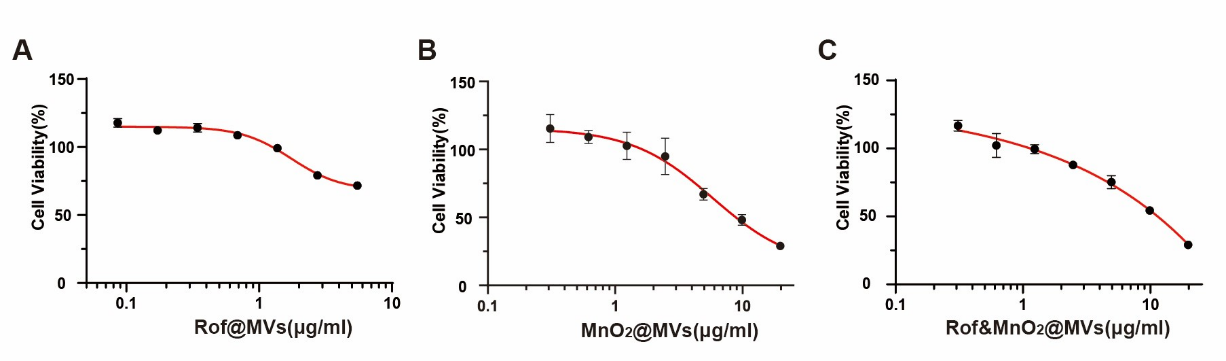


**Figure S6.** Cell viability assay of various nanoparticles. (A) Rof@MVs (the concentration of Rof). (B) MnO_2_@MVs (the concentration of MnO_2_). (C) Rof&MnO_2_@MVs (the concentration of MnO_2_ in the nanoparticles). The concentration ratio of Rof and MnO_2_ is constant 1:4 in Rof&MnO_2_@MVs. These data were manifested as mean±SD, n=6.


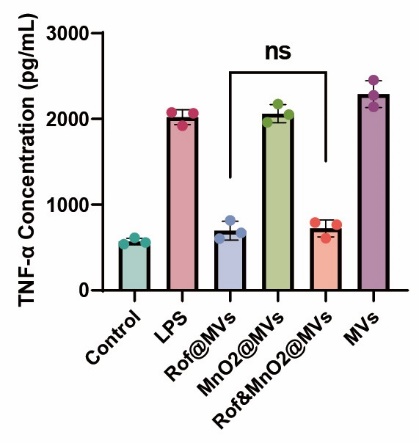


**Figure S7**. The efficacy of roflumilast and MnO_2_ in inhibiting TNF-α secretion from RAW264.7 (n=3). Rof@MVs (Rof, 2.5μg/mL), MnO_2_@MVs (MnO_2_, 5 μg/mL), Rof&MnO_2_@MVs (Rof, 2.5μg/mL; MnO_2_, 5 μg/mL) and MVs (62.5 μg /mL). The concentration ratio of roflumilast and MnO_2_ is 1:2.


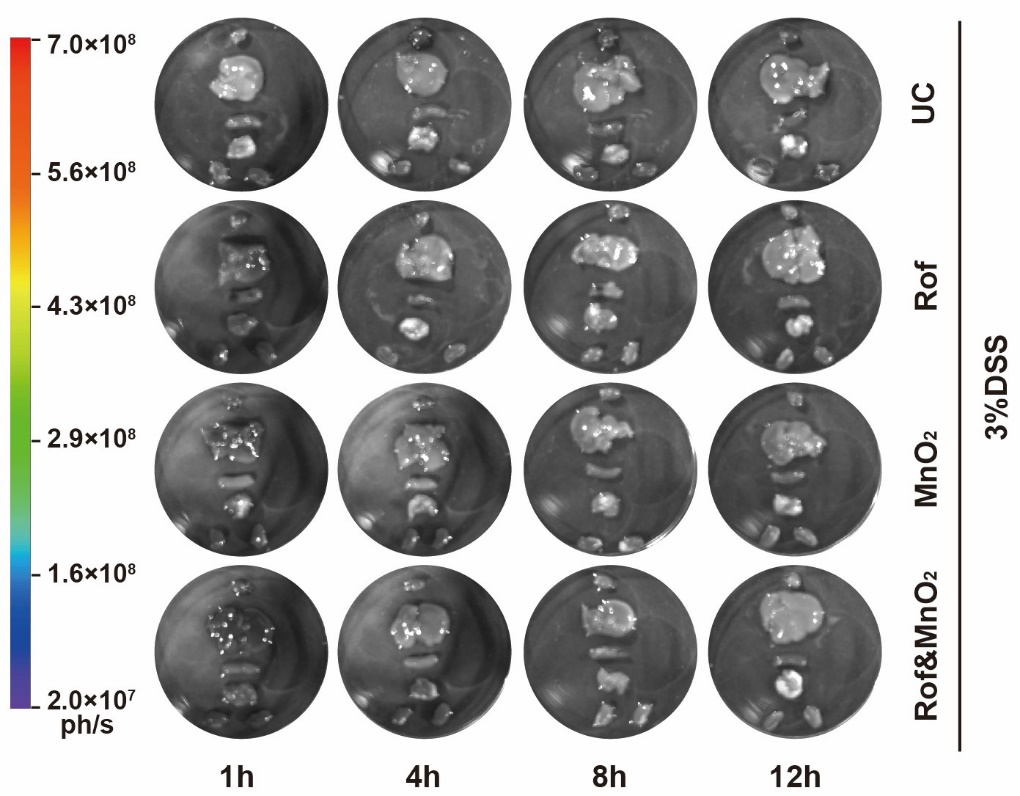


**Figure S8.** Biodistribution of nanoparticles in other organs. From top to bottom is heart, liver, spleen, lung and kidneys respectively in one picture. Abbreviations: Rof, Rof@MVs-FITC; MnO_2_, MnO_2_@ MVs-FITC; Rof&MnO_2_, Rof&MnO_2_@ MVs-FITC.


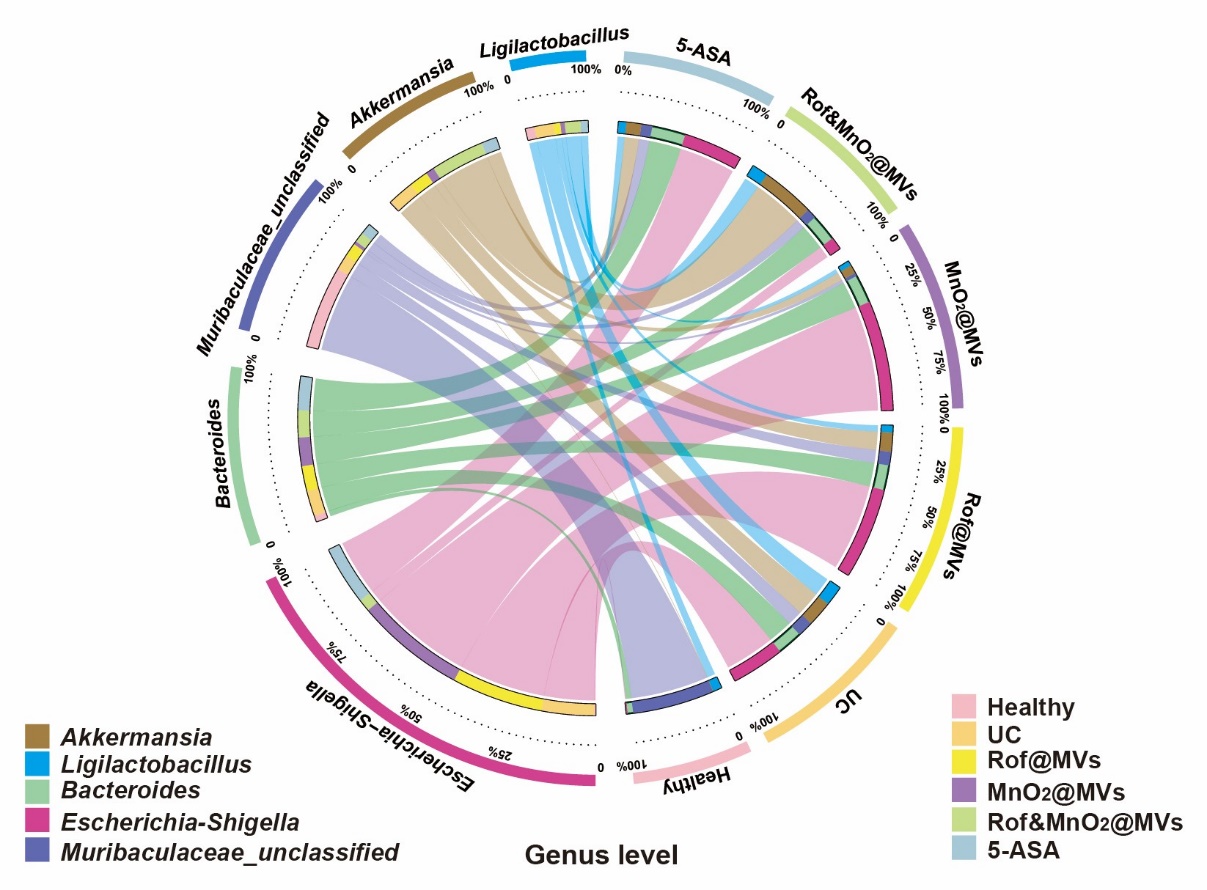


**Figure S9.** Circos of top 5 bacteria from different groups in genus level.


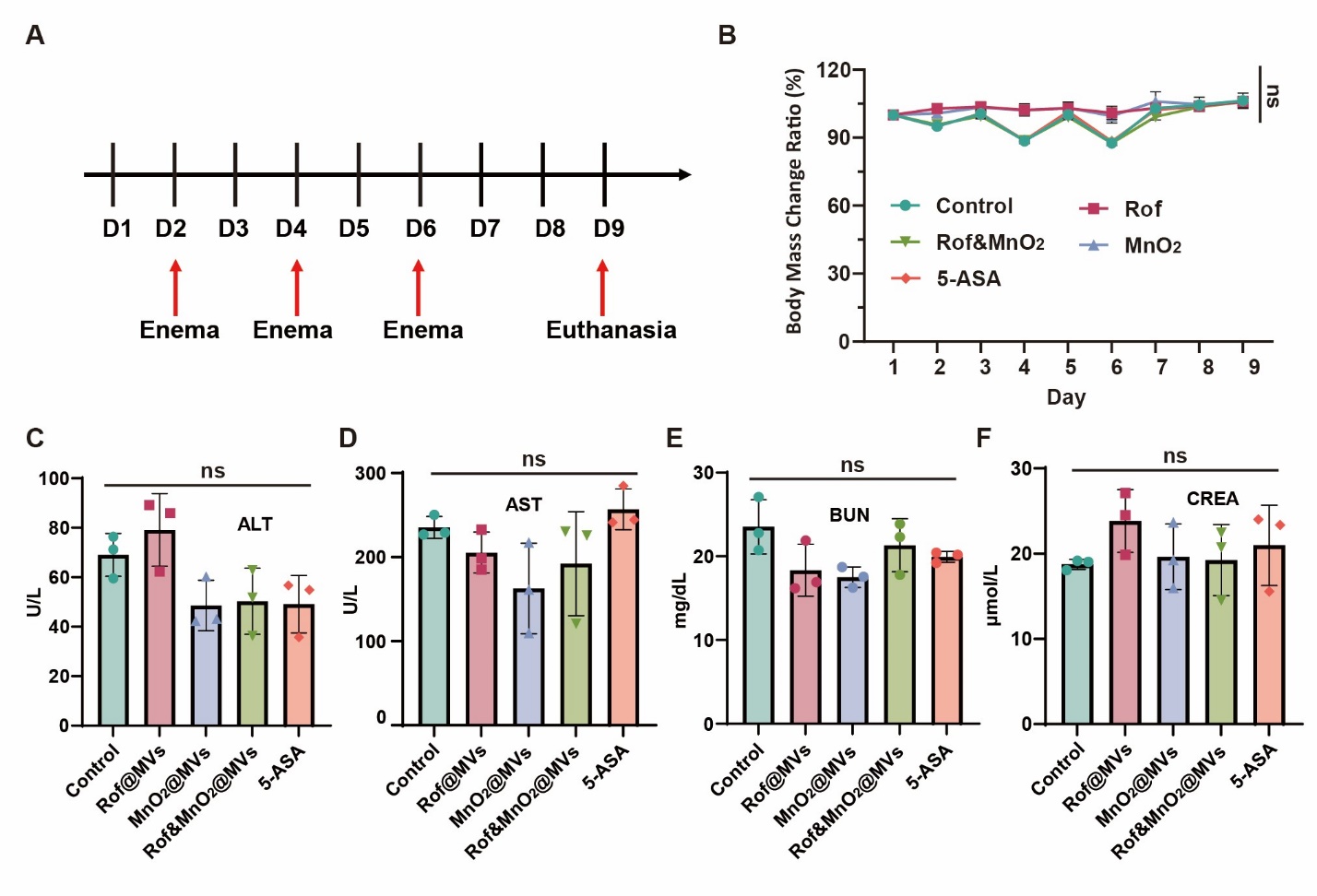


**Figure S10.** Biosafety assessment of MVs-based nanoparticles. (A) The scheme of experiment. Normal mice were used in this biocompatible examination. During this process, enema was performed on day 2 ,4 and 6 with Rof (Rof@MVs, roflumilast 1 mg/kg), MnO_2_ (MnO_2_@MVs MnO_2_, 4 mg /kg), Rof&MnO_2_ (Rof&MnO_2_@MVs, roflumilast, 1mg/kg and MnO_2_, 4 mg/kg) and 5-ASA (1.25 mg/kg). On day 9, mice were sacrificed and colon was collected for further analysis. (B) Dynamic body weight mass in different groups during the experiment (n=3). (C) ALT level in the serum (n=3). (D) AST level in the serum (n=3). (E) BUN level in the serum (n=3). (F) CREA level in the serum (n=3). Abbreviations: ALT, alanine transaminase; AST, aspartate transaminase; BUN, blood urea nitrogen; CREA, creatinine. These data were manifested as mean±SD. *p<0.05, **p<0.01, ***p<0.001, ****p<0.0001, ns (none significance). Data was statistically analyzed via one-way ANOVA multiple comparisons tests (Tukey’s test was used for comparison of multiple groups).


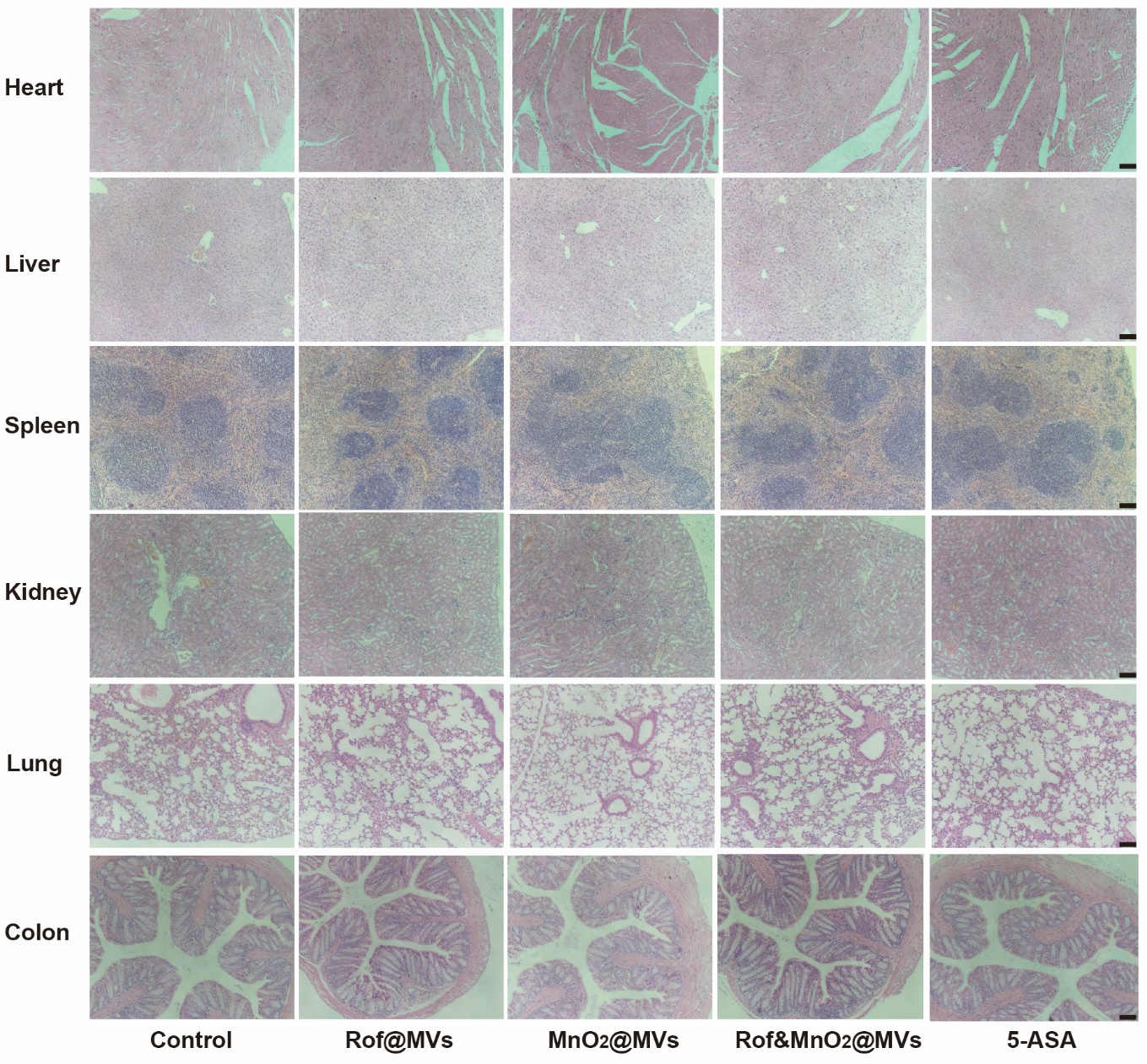
**Figure S11.** Representative images of H&E stain of organs in each group (scale bar 100 μm).
